# Allosteric modulation of nucleoporin assemblies by intrinsically disordered regions

**DOI:** 10.1126/sciadv.aax1836

**Published:** 2019-11-27

**Authors:** Bartlomiej Jan Blus, Junseock Koh, Aleksandra Krolak, Hyuk-Soo Seo, Elias Coutavas, Günter Blobel

**Affiliations:** 1 Laboratory of Cell Biology, Howard Hughes Medical Institute, The Rockefeller University, New York, NY 10065, USA.; 2 School of Biological Sciences, Seoul National University, Seoul, 08826, South Korea.; 3 Department of Cancer Biology, Dana-Farber Cancer Institute, Boston, MA 02215, USA.; 4 Department of Biological Chemistry and Molecular Pharmacology, Harvard Medical School, Boston, MA 02115, USA.

## Abstract

Intrinsically disordered regions (IDRs) of proteins are implicated in key macromolecular interactions. However, the molecular forces underlying IDR function within multicomponent assemblies remain elusive. By combining thermodynamic and structural data, we have discovered an allostery-based mechanism regulating the soluble core region of the nuclear pore complex (NPC) composed of nucleoporins Nup53, Nic96, and Nup157. We have identified distinct IDRs in Nup53 that are functionally coupled when binding to partner nucleoporins and karyopherins (Kaps) involved in NPC assembly and nucleocytoplasmic transport. We show that the Nup53·Kap121 complex forms an ensemble of structures that destabilize Nup53 hub interactions. Our study provides a molecular framework for understanding how disordered and folded domains communicate within macromolecular complexes.

## Teaser

Kap121 interacts with an intrinsically disordered region of Nup53 to allosterically modulate Nup53 binding to partner nucleoporins.

## INTRODUCTION

Proteins containing intrinsically disordered regions (IDRs) are widespread in the eukaryotic proteome and contribute to cellular regulation and signaling. Given their inherent flexibility and binding promiscuity, IDRs often mediate the formation of dynamic heterogeneous macromolecular complexes and phase-separated condensates, and their dysfunction has been associated with pathological conditions and disease (*1*, *2*). Although phenomenological studies of these assemblies are attracting increasing interest, it remains elusive how their conformational states and thereby biological function are modulated by IDRs, particularly in response to external signals. This is due, in part, to the challenges in quantifying intricate interaction networks and visualizing distinct conformations of IDR-driven macromolecular complexes at high resolution. Moreover, IDRs within multidomain eukaryotic proteins are often unstable and prone to aggregation and proteolytic degradation; by necessity, they have been routinely analyzed as minimal functional fragments, thus limiting our understanding of their full regulatory potential.

**Fig. 1:**
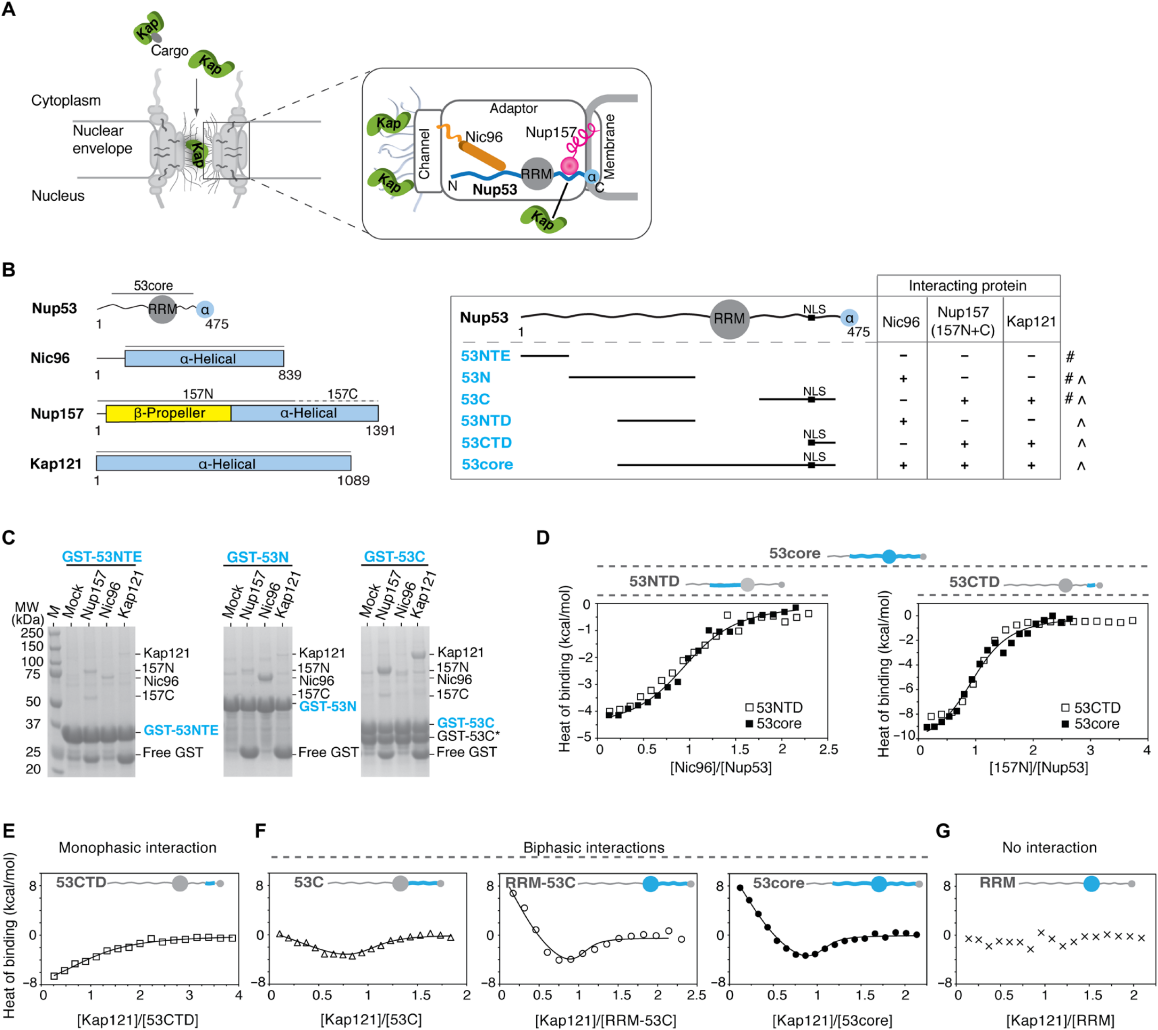
Nup53 uses distinct IDRs for binding to partner nups and Kap121. (**A**) A cross-sectional view of the NPC shows distinct nup subcomplexes linked by IDRs in adaptor nups (wavy lines). Kaps (green) facilitate NLS-mediated nuclear import. The zoomed-in section highlights the adaptor Nup157**·**Nup53**·**Nic96 complex that bridges the channel and membrane nup modules. **(B)** Domain architecture of Nup53, Nic96, Nup157, and Kap121 (left panel). Gray lines mark boundaries of the protein constructs used in this study. Table showing Nup53 fragments and the results of pull-down (#) and ITC (˄) binding assays (right panel). **(C)** Pull-downs using partially purified, GST-immobilized Nup53 fragments with Nic96, Nup157 (157N and 157C), or Kap121. Eluted GST**·**Nup53 complexes were visualized by SDS–polyacrylamide gel electrophoresis (PAGE) (M, molecular weight marker; GST-53C*, proteolytic truncation product). **(D)** ITC profiles for 53NTD-Nic96 and 53CTD-Nup157 interactions overlaid with Nic96 or 157N titrations with 53core. Integrated heat signals were analyzed by a single-site 1:1 binding model, yielding binding constants of 7.0 (± 2.2) × 10^5^ M^−1^, 9.9 (± 0.9) × 10^5^ M^−1^, 1.6 (± 0.2) × 10^6^ M^−1^, and 1.1 (± 0.3) × 10^6^ M^−1^ for 53NTD-Nic96, 53core-Nic96, 53CTD-Nup157, and 53core-Nup157 interactions, respectively. (**E** to **G**) ITC profiles for interactions of Kap121 (150 μM) with different Nup53 fragments (15 μM; blue). Monophasic binding data (E) were fitted with a single-site 1:1 binding model. The biphasic binding isotherms (F) were analyzed by either two-mode (53C) or multiple-equilibrium (RRM-53C and 53core) binding models (continuous lines). The best-fit parameters and the associated errors from global analysis are presented in Table 1. The RRM domain in Nup53 does not interact with Kap121 (G).

The nuclear pore complex (NPC) is a representative example for studying IDRs within an intact system, as it consists of disordered and structured regions that are essential to its function (*3*, *4*). Composed of ~30 different nucleoporins (nups), each NPC forms a cylindrical channel that orchestrates selective transport of macromolecules between the nucleus and cytoplasm (*5*–*7*). While most nups use their folded domains to arrange into specific subcomplexes within the NPC, the interactions within and between these modules are further mediated by short disordered motifs in linker or adaptor nups (*8*, *9*). In yeast, adaptor Nup53 uses distinct IDRs for binding to nups Nic96 and paralogous pairs of Nup157/Nup170 and Nup188/Nup192 (*10*, *11*). The intact Nup53 complex thus bridges the innermost region of the central transport channel and the outermost membrane-bound scaffold of the NPC (Fig. 1A) (*12*, *13*). In addition, Nup53 contains a nuclear localization signal (NLS) sequence known to interact with karyopherins (Kaps)—soluble transport factors implicated in NPC assembly and nucleocytoplasmic transport (*14*–*16*).

Thus, the Nup53 hub is a suitable model system for exploring (i) the role of IDRs within large macromolecular complexes and (ii) the effect of external stimuli, such as Kap binding, on the conformation of IDR-mediated complexes. To tackle this challenge, we characterized the interactions between Nup53 and its binding partners in pairs and in the assembled complex with and without the Kap using isothermal titration calorimetry (ITC). Last, we visualized Kap binding to this nup assembly by x-ray crystallography and electron microscopy (EM).

## RESULTS

### Discrete IDRs in Nup53 mediate interactions with nups and Kaps

We first identified regions in Nup53 that interact with Nic96, Nup157, and Kap121 (Fig. 1B and table S1) and established that these Nup53 fragments are highly disordered in solution (fig. S1, A to C). In agreement with previous studies using thermophilic and vertebrate homologs (*11*, *17*), we found that Nic96 interacts with the N-terminal disordered region of Nup53 (53N), whereas Nup157 (157N) or Kap121 specifically binds to the C-terminal IDR in Nup53 (53C) (Fig. 1C). Using ITC, we then mapped even shorter Nup53 fragments—53NTD and 53CTD—which bind to Nic96 and 157N with a stoichiometry ratio of 1:1 and binding constants of 7.0 (± 2.2) × 10^5^ M^−1^ and 1.6 (± 0.2) × 10^6^ M^−1^, respectively (fig. S1D). 53NTD and 53CTD contain intact binding sites for Nic96 and Nup157 because their ITC binding profiles are comparable to those of a near-intact Nup53 construct (53core) encompassing both regions (Fig. 1D and Table 1). We further found that 53CTD contains a peptide binding site for 157N (53CTDΔ; fig. S1E) and confirmed that the adjacent NLS motif binds to Kap121 with a stoichiometry ratio of 1:1 and a binding constant of 2.1 (± 0.7) × 10^5^ M^−1^, which is ~45-fold weaker when compared to the binding of a canonical NLS peptide (Fig. 1E and fig. S1E).

**Table 1:** Summary of ITC data. Detailed description of rows 1 to 5 is provided in Materials and Methods. ∆*G*° and *T*∆*S*° were determined by ∆*G*° = −*RT* ln*K* and *T*Δ*S* = Δ*H*° − Δ*G*°, where *R* and *T* are gas constant (1.99 cal mol^−1^ K^−1^) and temperature (288 K), respectively. The errors are the SDs obtained from the global fits, pertaining to uncertainties in both the model accuracy and measurement precision (i.e., model and experimental errors). (1) 53core dissociation profiles obtained at three protein concentrations were analyzed by a monomer-dimer equilibrium model. (2) 53C·Kap121 forward and reverse titrations, performed at various protein concentrations, were globally analyzed by a two-mode binding model, yielding thermodynamic constants for 1:1 and 2:1 (53C:Kap121) interactions in rows 2a and 2b. These parameters also describe interactions between monomeric 53core and Kap121. 53core·Kap121 forward and reverse titrations, performed at various protein concentrations, were globally analyzed by a multiple-equilibrium model using constants in rows 1, 2a, and 2b. The obtained parameters in 2c and 2d describe 2:1 and 2:2 interactions between 53core dimer and one and two molecules of Kap121, respectively. A_2_*B and A_2_B complexes differ in the oligomeric state of A (two monomers versus dimer). (3 and 4) Nic96·53core and Nup157·53core binding profiles were analyzed by a single-site 1:1 binding model. (5) Nic96 titrations into stoichiometric 53core:Kap121 or 53core:*Hs*Kapβ1 mixtures were analyzed by a single-site 1:1 model to obtain apparent *K*’s (*K_app_*) for 53core-Nic96 interactions in the presence of Kaps (rows 5a and 5a′). Titrations were globally analyzed by a multiple-equilibrium model to deconvolute Nic96 binding to monomeric and dimeric 53core**·**Kap121 complexes (rows 5b and 5c, respectively). Parameters describing Nic96 interactions with the monomeric 53core**·**Kap121 complex were independently obtained using a dimerization-deficient Nup53_mut_ fragment (table S1 and fig. S7, A and B). The subscript *i* in Kap121_i_ denotes the number of bound Kap molecules (*i* = 1 or 2).

**Note**	Interaction*	***K***	**∆*H*° (kcal/mol)**	**∆*G*° (kcal/mol)**	***T*∆*S*° (kcal/mol)**
1	2A ↔ A_2_	1.0 (± 0.1) × 10^4^ M^−1^	6.5 ± 0.2	−5.3 ± 0.1	11.8 ± 0.2
2a	A + B ↔ AB	4.2 (± 0.7) × 10^6^ M^−1^	−2.7 ± 0.1	−8.7 ± 0.1	6.0 ± 0.1
2b	2A + B ↔ A_2_*B	1.9 (± 0.5) × 10^11^ M^−2^	4.2 ± 0.7	−14.9 ± 0.2	19.1 ± 0.8
2c	A_2_ + B ↔ A_2_B	5.6 (± 0.8) × 10^7^ M^−1^	16.3 ± 1.4	−10.2 ± 0.1	26.5 ± 1.4
2d	A_2_ + 2B ↔ A_2_B_2_	3.9 (± 1.0) × 10^14^ M^−2^	−1.3 ± 0.4	−19.2 ± 0.1	17.9 ± 0.4
3	A + D ↔ AD	1.1 (± 0.3) × 10^6^ M^−1^	−10.1 ± 0.5	−8.0 ± 0.2	−2.1 ± 0.5
4	A + C ↔ AC	9.9 (± 0.9) × 10^5^ M^−1^	−4.4 ± 0.1	−7.9 ± 0.1	3.5 ± 0.1
5a	(AB)_app_ + C ↔ (AB)_app_C	1.3 (± 0.4) × 10^5^ M^−1^	−5.2 ± 0.6	−6.7 ± 0.2	1.5 ± 0.6
5a′	(AB′)_app_ + C ↔ (AB′)_app_C	1.0 (± 0.1) × 10^5^ M^−1^	−5.1 ± 0.4	−6.6 ± 0.1	1.5 ± 0.4
5b	AB + C ↔ ABC	2.0 (± 0.9) × 10^5^ M^−1^	−6.7 ± 1.7	−7.0 ± 0.3	0.3 ± 1.7
5c	A_2_B_i_ + C ↔ A_2_B_i_C	1.2 (± 0.2) × 10^5^ M^−1^	−2.5 ± 0.3	−6.7 ± 0.1	4.2 ± 0.3

*53core (A), Kap121/*Hs*Kapβ1 (B/B′), Nic96 (C), and 157N (D).

### Kap121 bivalently interacts with the C-terminal IDR in Nup53

Surprisingly, a complete Kap121 binding site in Nup53 extends beyond the NLS sequence. We observed that an ~80-residue-long, C-terminal IDR in Nup53 (53C) is protected by Kap121 from trypsin digestion and that a Nup53 fragment lacking the NLS also binds Kap121 (fig. S2, A and B). In contrast to the monophasic Kap121-53CTD binding profile (Fig. 1E), Kap121 interaction with 53C is biphasic, suggestive of the presence of at least two distinct binding modes (Fig. 1F) (*18*). This biphasic ITC thermogram is further enhanced, particularly in the first phase of titration, for the N-terminally extended Nup53 fragments—RRM-53C and 53core—containing the conserved RNA recognition motif (RRM) domain (Fig. 1F). As the RRM-53C and 53core binding isotherms are thermodynamically equivalent (fig. S2C), this suggests that the N-terminal IDR in 53core (53NTD) does not affect 53core binding to Kap121, whereas the substantial contribution of the RRM to these interactions is indirect, as the RRM fragment alone does not bind Kap121 (Fig. 1G).

To uncover the molecular basis for the apparent complexity of Kap121-Nup53 interactions, we took a two-step approach in which we first analyzed the direct binding of Kap121 to 53C and then quantified the effect of the RRM domain on these interactions using a near-intact 53core fragment. For nonmonophasic binding isotherms, it is possible to distinguish heat signatures corresponding to different protein complexes by combining the results into global analysis linked to a nonlinear least squares algorithm (*19*). In global analysis, ITC experiments are performed at various protein concentrations to perturb the distribution of distinct states of a macromolecular complex to selectively enhance heat signal from a specific state. Then, these multiple titrations are simultaneously fitted to a model with a minimal set of variable parameters that accurately reflect biochemical and structural features of the studied interactions (see Materials and Methods for details).

**Fig. 2:**
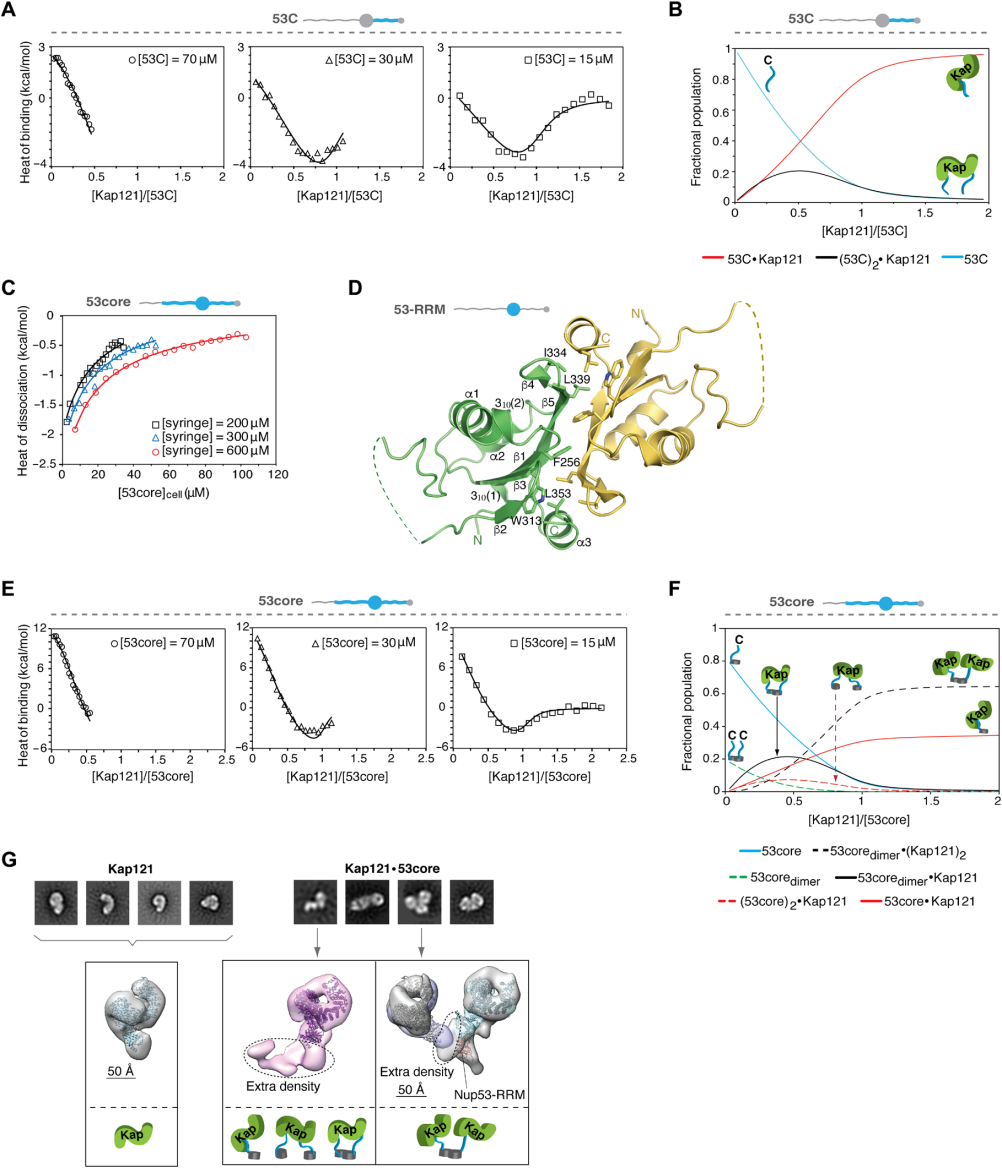
Nup53 RRM and IDR domains are coupled when interacting with Kaps. (**A**) ITC profiles for 53C-Kap121 interactions at three different 53C concentrations. The isotherms were globally analyzed together with the reverse titration in fig. S2D by a two-mode binding model (continuous lines; best-fit parameters with SDs are reported in Table 1). **(B)** Population distribution of 1:1 and 2:1 53C**·**Kap121 complexes is displayed as a function of [Kap121]/[53C] molar ratio. **(C)** 53core monomer-dimer equilibrium at different concentrations analyzed by ITC using a single-step dissociation model (continuous lines). **(D)** Crystal structure of the yeast Nup53 RRM homodimer determined at a resolution of 1.75 Å. Individual monomers are shown with secondary structure elements. Key hydrophobic residues contributing to the interface are highlighted in the green monomer. Regions not observed in the electron density map are shown as dashed lines. **(E)** ITC profiles for 53core-Kap121 interactions at three 53core concentrations were globally analyzed by a multiple-equilibrium model (continuous lines; best-fit parameters with SDs are reported in Table 1). **(F)** Population distribution of the 53core**·**Kap121 complex is shown as a function of [Kap121]/[53core] molar ratio. **(G)** Negative-stain EM (NS-EM) analysis of Kap121 and its complex with 53core. Representative 2D class averages and 3D reconstitution of free Kap121 and the two distinct conformations of the 53core**·**Kap121 complex are displayed with fitted crystal structures of Kap121 (PDB code: 3W3T). The monomeric state of the 53core**·**Kap121 complex (middle panel) shows extra density that likely represents either a 53core monomer, dimer, or an average of the two conformations, as determined by ITC. To assign orientation of the two Kap molecules within the dimeric state of the complex (right panel), the experimental NS-EM density map for free Kap121 (purple and cyan) was fitted into each of the arms of the structure. The RRM domain of Nup53 (PDB code: 5UAZ) was docked into the stemlike portion of the structure.

Using this strategy, we found that 53C binds Kap121 with stoichiometry ratios of 2:1 and 1:1 and binding constants of 1.9 (± 0.5) × 10^11^ M^−2^ and 4.2 (± 0.7) × 10^6^ M^−1^, respectively (Fig. 2A, Table 1, and fig. S2D). Using the measured binding constants, we found that both Kap121·53C conformations are present at substoichiometric amounts of Kap121, whereas the 1:1 complex predominates as the Kap concentration increases (Fig. 2B). At saturating conditions, the binding of Kap121 to 53C is thus ~25-fold tighter than its interactions with the NLS sequence in Nup53. To further probe the molecular nature of 53C-Kap121 interactions, we performed competition assays with other binding partners of Kaps. We found that a canonical NLS sequence effectively competes with 53C for Kap121 binding (fig. S2E), while a peptide derived from Nup58 containing multiple Phe-Gly (FG) motifs shows a weaker but statistically meaningful effect (fig. S2F). These results suggest that 53C makes extensive contacts with Kap121 that involve both the concave (NLS binding site) and convex (FG binding site) binding surfaces of Kap (*20*, *21*).

### Nup53 dimerizes via its RRM domain

Because vertebrate Nup53 dimerizes via the RRM domain (*22*, *23*), we reasoned that dimerization of the yeast Nup53 could contribute to the pronounced biphasic profile of the 53core-Kap121 titration. We found that 53core dimerizes in solution (Fig. 2C) and solved a crystal structure of the Nup53 RRM dimerization domain at a resolution of 1.75 Å (Fig. 2D and table S2). Like the vertebrate homologs, the yeast RRM forms an antiparallel homodimer, with each monomer adopting essentially the same structure (core root mean square deviation of the Cα positions, ~0.8 Å). The dimer interface is mainly stabilized by hydrophobic interactions (Fig. 2D and fig. S3A), and mutations of the two conserved residues, Phe^256^ and Trp^313^, completely block RRM dimerization in a vertebrate homolog of Nup53 (*23*). Notably, the remaining residues involved in the dimer interface are conserved to a lesser degree between the yeast and human RRMs, suggesting potential differences in Nup53 dimer stability across various species (fig. S3, B and C).

### An ensemble of Nup53·Kap121 complexes is driven by Nup53 dimerization

We then globally analyzed the 53core·Kap121 isotherms accounting for the dimerization of 53core and the interactions of 53core monomers and dimers with Kap121 (Fig. 2E and Table 1). In our fitting scheme, we used the binding constants derived from the analysis of 53C-Kap121 titrations to describe 53core monomer interactions with Kap121 (1:1 and 2:1 complexes), which is justified by our systematic analysis of different model assumptions (fig. S4, A to D; see Materials and Methods for details). The global analysis yielded the binding parameters for the 53core dimer interactions with Kap121 (Table 1). We found that 53core interacts with Kap121 predominantly as a dimer at substoichiometric amounts of Kap. The affinity of this interaction, 5.6 (± 0.8) × 10^7^ M^−1^, is approximately two orders of magnitude higher than that determined for Kap121 and the two C-terminal IDRs (53C) in Nup53, 4.4 (± 1.2) × 10^5^ M^−1^ per IDR binding (i.e., square root of the binding constant in row 2b in Table 1) (Fig. 2B). When the RRM domain in Nup53 is replaced with the constitutive glutathione *S-*transferase (GST) dimer, the pronounced biphasic binding profile is reduced to the levels observed for Kap121 titrations with 53C, which lacks the RRM (fig. S4E). This suggests that the RRM dimer uniquely positions the two C-terminal IDRs to maximize a local concentration effect (i.e., reduction of dimensionality) (*24*) and therefore promotes cooperative high-affinity 53core-Kap121 interactions. As Kap121 concentration increases, complexes with one molecule of Kap bound per a disordered region of 53core become predominant [i.e., 53core_dimer_:(Kap121)_2_ and 53core:Kap121] (Fig. 2F). Given that the binding of a human Kapβ1 fragment to 53core is also biphasic (fig. S4F), our results collectively point to a potentially conserved mechanism of Nup53 interactions with Kaps.

### Molecular architecture of Nup53·Kap121 complexes

To gain structural insights into this process, we examined Kap121 or its complex with 53core by negative-stain EM (NS-EM). In the Kap121 sample, we found a single class of molecules with a characteristic S-shaped architecture common to all Kapβs (Fig. 2G) (*25*). In contrast, the 53core·Kap121 complex encompasses either one or two Kap molecules (Fig. 2G and fig. S5A). The “monomeric” conformation of the complex contains Kap121 with an additional extended density region, likely corresponding to the bound 53core monomer or dimer. The “dimeric” conformation of the 53core·Kap121 complex resembles an asymmetric letter Y, with the two bulky “arms” of the molecule connected by a density emanating from a stemlike region (Fig. 2G). Docking Kap121 into this model revealed its preferential orientation within each arm of the complex. However, it is unlikely that either Kap121 or the disordered regions in 53core account for the distal stem region of the structure. Instead, this density is presumably occupied by the RRM, as evidenced by preferential fitting of our crystal structure into this region. Furthermore, the NS-EM structure of Kap121 bound to a Nup53 fragment lacking the RRM domain (53C) contains a single copy of the Kap without a connecting stemlike density (fig. S5B).

### Interactions within the Nup53 hub are allosterically modulated by Kaps

**Fig. 3:**
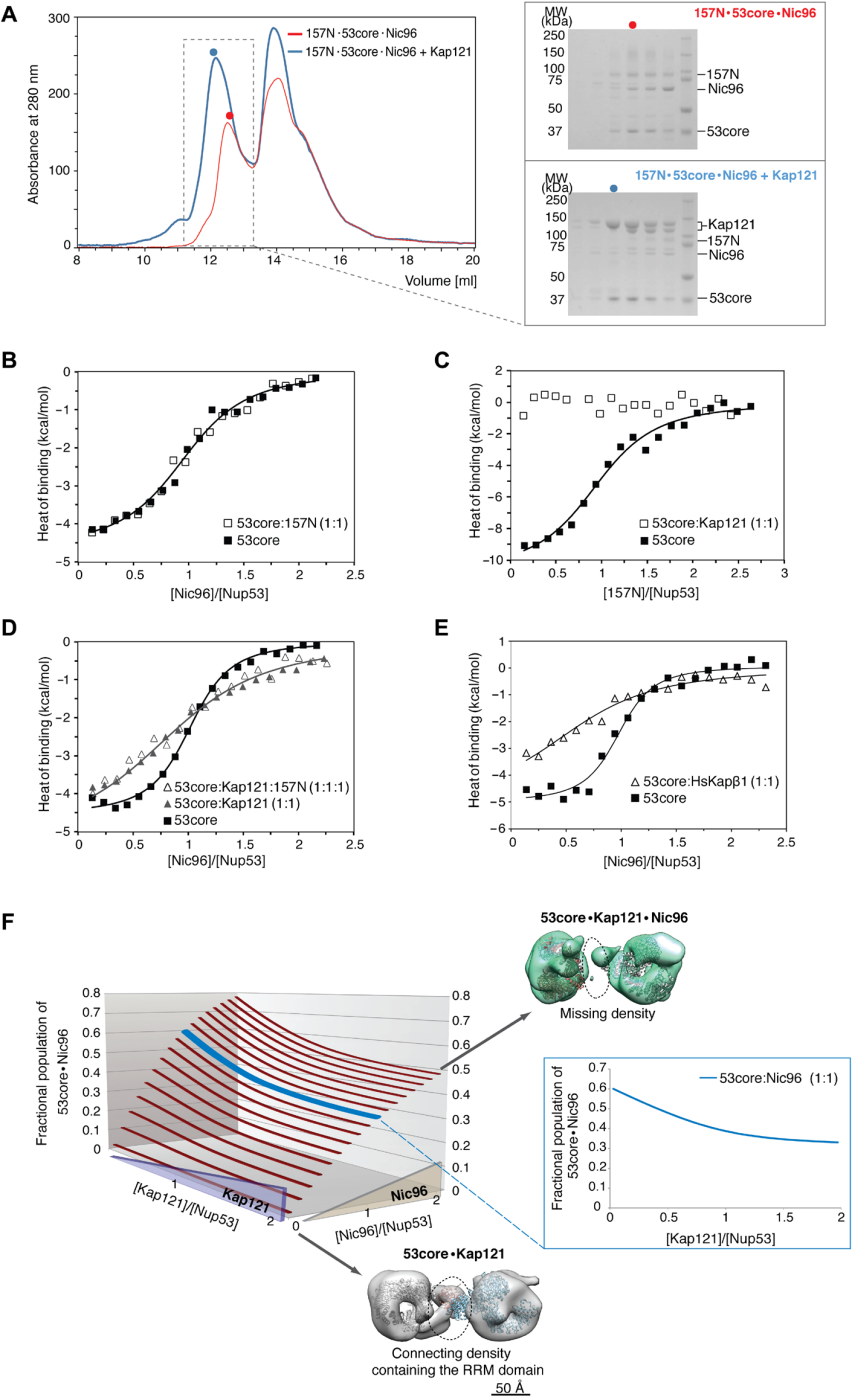
Kap121 allosterically destabilizes ternary 157N·53core·Nic96 complex. (**A**) SEC elution profiles of ternary 157N**·**53core**·**Nic96 complex alone (red trace) and preincubated with Kap121 (blue trace). Colored dots denote elution peak maxima, and dashed line rectangle represents the range of elution fractions visualized by SDS-PAGE on the right. (**B** to **E**) ITC profiles for interactions between (B) 53core and Nic96 in the absence or presence of 157N, (C) 53core and 157N in the absence or presence of Kap121, (D) Nic96 and 53core or equimolar 53core:Kap121 or 157N:53core:Kap121 mixtures, and (E) Nic96 and 53core or its equimolar mixture with *Hs*Kapβ1. The ITC binding profiles were analyzed by a single-site 1:1 binding model (continuous lines; best-fit parameters with SDs are reported in Table 1). (**F**) 3D population distribution plot of the 53core**·**Kap121**·**Nic96 complex. Increasing Kap121 concentration gradually destabilizes the 53core-Nic96 interactions—at a 1:1 ratio of 53core and Nic96, the fractional population of the 53core**·**Nic96 complex decreases from ~0.6 to 0.3 as the [Kap121]/[Nup53] molar ratio increases from 0 to 2. NS-EM models of 53core**·**Kap121 (gray) and 53core**·**Kap121**·**Nic96 (green) at a stoichiometry ratio of 2:2 are shown in top view with fitted crystal structures of Kap121 and the Nup53 RRM domain. Dashed ovals denote electron density differences between binary and ternary complex.

Having established that Nup53 uses distinct mechanisms to interact with nups and Kaps, we explored how these proteins communicate with one another to regulate the conformational state of the assembled complex. To do so, we reconstituted various Nup53 complexes using size exclusion chromatography (SEC) in the absence or presence of Kap121 (Fig. 3A and fig. S6). Consistent with the formation of a ternary nup complex, the up-shifted peak elution fractions contained 157N, 53core, and Nic96. Surprisingly, incubation of Kap121 with the preassembled 157N·53core·Nic96 complex and subsequent separation of high–molecular weight fractions revealed that 53core no longer associates with 157N and predominately coelutes with Kap121 and trace amounts of Nic96 (Fig. 3A).

To confirm and quantify these observations, we designed a series of ITC titrations using different combinations of up to three nups and/or Kap121. First, we confirmed the formation of a ternary nup complex mediated by 53core and further observed that the interactions of 96C and 157N within this assembly are independent of each other (Fig. 3B). Consistent with SEC results, there is also no detectable binding of 157N to 53core in the presence of Kap121 (Fig. 3C). A titration of the 53core:Kap121 mixture with 96C yielded a broader binding isotherm than the two-component titration (Fig. 3D). As a consequence, the measured binding affinity of 96C to 53core was approximately eightfold weaker, with an apparent binding constant of 1.3 (± 0.4) × 10^5^ M^−1^ in the presence of Kap121 (Table 1). Using a dimerization-deficient Nup53 fragment, we further show that Nic96 binds Nup53 monomers and dimers five- and eightfold weaker, respectively, in the presence of Kap121 (Table 1 and fig. S7, A and B). Because Kap121 and Nic96 do not interact with each other (fig. S7C) and they bind to distinct, widely separated, sites in 53core (C- and N-terminal IDRs, respectively), the observed destabilization effect is exclusively allosteric. A human Kapβ1 fragment had a comparable allosteric effect on these nup interactions (Fig. 3E), reducing the apparent binding affinity of Nic96 to 53core by ~10-fold (Table 1). Last, the four-component titration (96C into 157N·53core·Kap121) shows no further effect from 157N, as expected from the complete exclusion of 157N from 53core by Kap121 (Fig. 3D).

These results can be quantitatively represented in a three-dimensional (3D) plot (Fig. 3F). For instance, at a Nic96:53core ratio of 1:1, fractional occupancy of this complex decreases from ~0.6 to 0.3 as the Kap121:53core ratio increases from 0 to 2. To further visualize how allostery may regulate Nup53 binding to Kap121 and Nic96, we imaged the ternary Nic96·53core·Kap121 complex by NS-EM (Fig. 3F and fig. S8). In comparison with the “dimeric” state of the 53core·Kap121 complex, the corresponding conformation of the ternary complex is destabilized, lacking the density connecting the Kaps and the stemlike structure. Furthermore, we were unable to identify the precise location of the bound Nic96 in our 3D model, suggesting that its binding within the ternary complex is conformationally dynamic.

## DISCUSSION

**Fig. 4:**
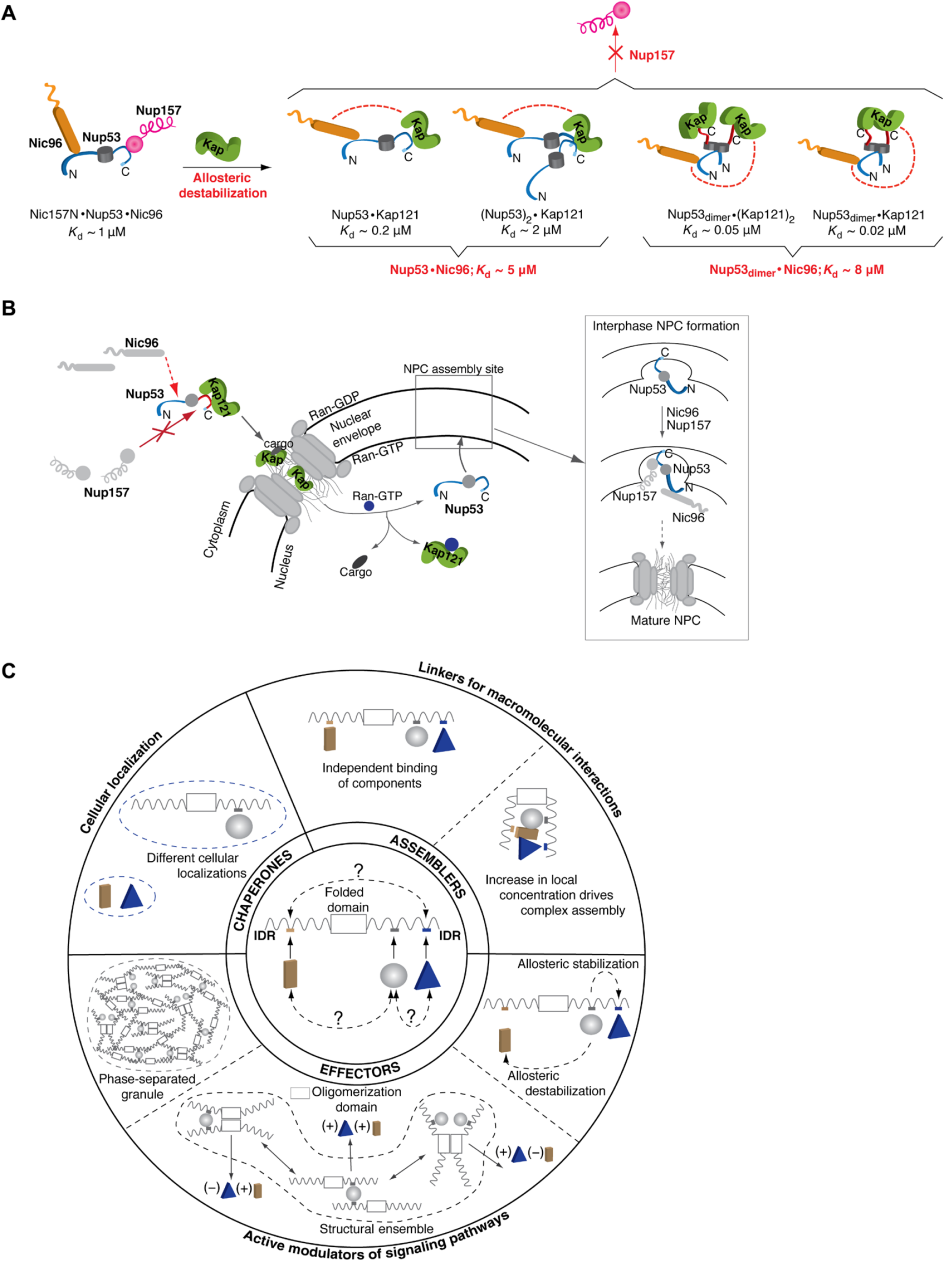
Karyopherins modulate molecular architecture of nucleoporin assemblies. (**A**) Schematic representation of Nup53 hub interactions. The ternary complex containing Nup157, Nup53, and Nic96 is destabilized by Kap121 binding to the Nup53 C-terminal IDR. Kap121 allosterically reduces Nic96 affinity for the monomeric and dimeric conformations of the complex. Dissociation constants (*K*_d_s) for the different complexes were derived from Table 1. (**B**) Proposed model for allostery-driven Nup53 hub assembly in yeast. Kap121 binds to a cytoplasmic pool of Nup53 to destabilize its interactions with Nic96 and Nup157. In the nucleus, Ran-GTP releases Nup53. At the nuclear envelope, Nup53 sequentially recruits other adaptor nucleoporins. In the final step of NPC assembly, Nic96 recruits channel nucleoporins. (**C**) Classification of IDR-mediated interactions within macromolecular complexes. A hub protein consisting of a folded domain and IDR binding sites for three interacting partners is shown in the center. Different binding scenarios are considered in increasing order of complexity. By binding to chaperones (top left), IDRs target proteins to particular subcellular locations. As assemblers, IDRs independently recruit macromolecules or promote avidity-driven interactions. As effectors, IDRs are often regulated by cooperative binding and/or allostery, actively modulating biological activity of macromolecular complexes or signaling pathways. Coupling of folded and IDR domains may promote the formation of a structural ensemble or a phase-separated granule (dashed lines).

Allosteric regulation mediated by IDRs was originally conceived in a thermodynamic framework (*26*, *27*) and observed in vivo (*28*) and in vitro using minimal functional domains of macromolecular systems (*29*, *30*). Here, we demonstrate IDR-mediated allostery in a large, near-intact, multicomponent system (Fig. 4A). Earlier studies have suggested that conformational transitions (e.g., disorder to order) may propagate among the IDRs and folded regions, thus driving their molecular coupling (*26*). Here, we show that the C-terminal IDR(s) and the RRM domain in Nup53 adopt a unique conformation within the 53core**·**Kap121 complex, allosterically restricting Nup53 interactions with its partner nups. Given a key role of a Kap/RanGTP gradient in regulating stepwise assembly of the Nup53 subcomplex into the NPC (*16*, *17*, *31*), we envision that Kaps may impede Nup53 interactions with other nups in the cytoplasm (Fig. 4B). Notably, mutations in Kaps/Ran can cause NPC assembly defects that manifest in cytoplasmic accumulation of NPC intermediates containing Nup53 binding partners Nic96 and Nup170 (Nup157 paralog) (*31*, *32*).

It is also feasible that allostery may contribute to regulating the entire NPC, particularly during increased transport flux and/or translocation of large cargo (e.g., ribosomal subunits and viral capsids) decorated with numerous Kaps. Given that Nup53 hub connects to the central channel, Kap-induced flexibility of the Nup53 interactomes could accommodate previously proposed structural changes in the central transport channel regulated by Kapβ1 (*33*, *34*). Although the molecular details of these long-range nup rearrangements have not been directly demonstrated, structural differences between native NPCs and those exhibiting reduced transport activities have been observed by cryo–electron tomography (cryo–ET) (*35*). Given the rapid progress in visualizing NPCs at increasingly higher resolution by cryo–ET (*36*–*39*), future reconstitutions coupled with systematic titrations of Kaps may provide further insights into the effect of transport factors on NPC architecture. Together with the available structural and biophysical data, such as ours, these studies may eventually lead to constructing a “functional” map of the NPC that would provide a more complete understanding of the transport mechanism. A similar approach has been taken to elucidate the molecular details of a potassium channel opening from an ensemble of its closed conformations (*40*).

Although most regulatory proteins segregate the binding sites for partner molecules into distinct folded and disordered domains, our study demonstrates that these distinct modules can be functionally coupled to facilitate allosteric communication among even distal members of a larger assembly (Fig. 4C). Our findings are likely not restricted to protein complexes with clearly defined stoichiometries, but they may also be relevant to IDR-driven liquid-liquid phase separation (LLPS) involving a multitude of transient interactions, such as P-bodies, stress granules, and super-enhancer–induced transcriptional condensates (*41*). It is currently unclear what molecular forces are behind LLPS and whether this process is proceeded by transitional states with more defined stoichiometries. We envision that our methodology could, in principle, reveal how different concentrations of IDR-containing macromolecules affect their interaction properties below the concentration threshold for phase separation and provide clues to pre-LLPS intermediates that drive the transition. In addition, ITC could be used to characterize protein mutants that lack the ability to phase separate, and thus help unravel key determinants for LLPS.

## MATERIALS AND METHODS

### Protein expression and purification

The DNA fragments corresponding to Nup53, Nic96, Nup157, and Kap121 proteins (all from *Saccharomyces cerevisiae*) as well as the human Nup35 RRM domain were generated by polymerase chain reaction from genomic DNA and cloned into modified pGEX-4T1 or pNIC28-Bsa4 vectors (Addgene) using ligation-independent cloning (*42*, *43*). The resulting fusion proteins contained N-terminal GST- or SUMO-His_6_ tags, cleavable with the tobacco etch virus (TEV) protease. Proteins were expressed in *Escherichia coli* BL21-CodonPlus(DE3)-RIL cells (Stratagene) following induction at OD_600nm_ (optical density at 600 nm) of 0.8 with 0.5 mM isopropyl-β-d-thiogalactopyranoside (IPTG) at 18°C for 12 to 16 hours in LB medium containing chloramphenicol (34 mg/liter) and ampicillin (100 mg/liter) or kanamycin (50 mg/liter). Cells were lysed using a cell disruptor (AVESTIN) in a buffer containing 20 mM Hepes (pH 7.5), 300 mM NaCl, 0.5 mM EDTA, 5% (v/v) glycerol, and 3 mM dithiothreitol (DTT; lysis buffer for GST-tagged proteins) or 20 mM Hepes (pH 7.5), 300 mM NaCl, 20 mM imidazole, 5% (v/v) glycerol, and 5 mM β-mercaptoethanol (BME; lysis buffer for His-tagged proteins). All lysis buffers were complemented with 1 mM benzamidine and phenylmethylsulfonyl fluoride as well as bovine lung aprotinin and leupeptin (1 μg/ml; Sigma-Aldrich). After centrifugation at 30,000*g* for 45 min, cleared supernatants were purified by standard chromatography techniques described below. A dimerization-deficient Nup53_mut_ fragment (W256E, W313E) was generated using the QuikChange Mutagenesis Kit (Stratagene). The details of the expression constructs are shown in table S1. *Hs*Kapβ1 and *Rn*Nup58 FG-repeat constructs were generated, expressed, and purified as previously described (*34*).

#### Purification of the yeast Nup53 and human Nup35 fragments

GST-tagged Nup53 lysates were loaded on a GSTrap column (GE Healthcare) and washed with the GST lysis buffer for ~75 column volumes. Proteins were eluted with 20 mM Hepes (pH 7.5), 100 mM NaCl, 5% glycerol, and 3 mM DTT supplemented with 15 mM reduced glutathione (Sigma-Aldrich). Following GST tag removal by TEV protease cleavage for 12 to 16 hours at 4°C, the Nup53 fragments were further purified over a HiTrap SP column (GE Healthcare) by gradually increasing ionic strength of the GST lysis buffer (corrected to pH 7.0) via a salt gradient from 100 to 600 mM NaCl. Last, proteins were purified over a HiLoad Superdex 200 26/60 gel filtration column (GE Healthcare) equilibrated with 20 mM Hepes (pH 7.5), 200 mM NaCl, and 3 mM DTT (protein storage buffer), concentrated to 5 to 15 mg/ml, flash-frozen in liquid nitrogen, and stored at −80°C.

#### Purification of Nic96 and Nup157

GST-tagged Nic96 and Nup157 fragments were purified using a previously described protocol (*44*). Briefly, proteins were affinity-purified over a GSTrap column, cleaved by TEV protease overnight at 4°C, and finally purified in the storage buffer using a Superdex 200 26/60 gel filtration column. As a last step, proteins were concentrated to 15 to 25 mg/ml, flash-frozen in liquid nitrogen, and stored at −80°C.

### Purification of Kap121

Cell lysates containing full-length Kap121 were applied to a HisTrap HP column (GE Healthcare) and washed with the His lysis buffer for ~15 column volumes, followed by a secondary wash with lysis buffer supplemented with 500 mM NaCl. Last, His-Kap121 was eluted with 20 mM Hepes (pH 7.5), 100 mM NaCl, 250 mM imidazole, 5% (v/v) glycerol, and 5 mM BME. After TEV protease cleavage overnight at 4°C, affinity-purified Kap121 was loaded onto the HiTrap Q column (GE Healthcare) and purified via a salt gradient from 100 to 600 mM NaCl. Kap121 was finally purified in a storage buffer using the Superdex 200 16/60 gel filtration column, concentrated to 25 to 30 mg/ml, flash-frozen in liquid nitrogen, and stored at −80°C.

### Pull-down and analytical SEC binding assays

Pull-down assays were performed in 20 mM Hepes (pH 7.5), 150 mM NaCl, and 3 mM DTT. Purified bait proteins Nic96, Nup157 (157N and 157C), or Kap121 (full-length) were each added in approximately 1.5 molar excess to ~10 μg of partially purified GST-tagged Nup53 fragments immobilized on 50 μl of packed glutathione-Sepharose 4B beads (GE Healthcare) and incubated for 1 hour at 4°C. Beads were spun down at 500*g* for 5 min and washed three times with 0.5 ml of the binding buffer. Bound proteins were eluted in SDS sample buffer and analyzed by SDS–polyacrylamide gel electrophoresis (PAGE) and Coomassie brilliant blue staining.

Binary and ternary nup complex reconstitutions by SEC were performed in the pull-down buffer using a Superdex 200 10/300 GL analytical gel filtration column (GE Healthcare). Different combinations of Nic96, Nup157 (157N), and Nup53 (53core) were incubated at equimolar ratios (50 μM each) for 1 hour at 4°C alone or in the presence of Kap121 (50 μM) before being applied to the column. High–molecular weight protein complexes were efficiently separated from the unbound proteins (Fig. 3A and fig. S6). For estimating hydrodynamic dimensions of the Nup53 fragments (53core, 53NTD, and 53C), purified proteins were applied to a Superdex 200 Increase 10/300 GL column (GE Healthcare) equilibrated with a pull-down buffer and previously calibrated with protein molecular weight markers (Bio-Rad). The N- and C-terminal Nup53 fragments are natively unfolded in solution as indicated by the disorder prediction algorithm and their circular dichroism (CD) spectra (fig. S1, A to C).

### Isothermal titration calorimetry

#### Introduction

ITC is a biophysical technique used to quantify biomolecular interactions. By directly measuring heat released or absorbed during a binding event, ITC can be used to determine a complete set of thermodynamic parameters (stoichiometry, binding constant, and enthalpy) in a single experiment. In principle, ITC is not limited to a particular type of a molecular interaction and does not require labeling of the interacting components, allowing the study of macromolecular complexes of any size in their native state. As ITC directly measures an incremental heat signal for each injection of a titrant (*45*), it is particularly suited for detecting concentration-dependent processes, including oligomerization, cooperativity, and/or multiple binding modes (e.g., interactions with different stoichiometries), provided that these processes exhibit distinct enthalpy changes (*46*, *47*). In contrast to the most common 1:1 interaction between biomolecules, the aforementioned phenomena typically manifest as nonmonophasic isotherms that need to be analyzed by a model containing multiple binding equilibria (e.g., 53core·Kap121 titrations). The contribution of individual events to a nonmonophasic isotherm can be further enhanced or diminished by changing the total protein concentration used in the experiment and/or reversing direction of the titration. Then, these isotherms are analyzed simultaneously at various protein concentrations (i.e., global analysis) with a suitable mathematical model to extract thermodynamic parameters describing these multiple equilibria (see Table 1). The obtained parameters can, in turn, be used to calculate population distribution of distinct complexes, providing a comprehensive view into how an ensemble of various molecular states is distributed as a function of protein concentration (e.g., Figs. 2, B and F, and 3F). Using this methodology, we were able to systematically characterize multiple equilibria describing interactions among up to four macromolecular components (three nups and a Kap).

#### Sample preparation and experimental conditions

Samples for the ITC experiments were dialyzed extensively (three changes, >24 hours total) against the ITC binding buffer [20 mM Hepes (pH 7.5), 150 mM NaCl, 0.5 mM tris(2-carboxyethyl)phosphine (TCEP)] using MINI dialysis devices with a molecular weight cutoff of 2.0 or 3.5 kDa (Thermo Scientific). After dialysis, proteins were filtered (0.22 μm) and centrifuged, before determining their concentration by ultraviolet absorbance at 280 nm. For the titrations, samples were loaded into a 96-deepwell plate with three consecutive wells filled for a single titration—samples for the reaction cell, the injection syringe, and the binding buffer for pre-rinsing the reaction cell. The fourth plate well was used to recover the reaction mixture at the completion of each titration for visual inspection of potential protein precipitation. All titrations for global analysis were performed using an automated MicroCal Auto-iTC_200_ instrument (GE Healthcare) at 15°C unless indicated otherwise. Titrations were performed at multiple protein concentrations as indicated in a figure, and each titration was repeated at least three times. In an ITC experiment, there are uncertainties regarding the effective protein concentration that arise from sample dilution by residual buffer in the ITC transfer tubing system and in the reaction cell, inaccuracies in the predicted extinction coefficients, and possible aggregation of the interacting macromolecules. Therefore, an activity term correcting the measured protein concentration was introduced to account for these effects. Typically, different purification batches of the same protein were active in the range of 75 to 100%.

#### ITC data analysis (global analysis and error statistics)

The formation of a single type of a complex (designated *S*) from two reactants is defined by the standard ITC equation (*48*) for the heat of the *i*th injection (*q_p,i_*) that takes into account the volume displacement in the reaction cell

$$q_{p,i}=\frac{\Delta H_{s}{}^{\circ}V_{\text{cell}}}{{\left[R\right]}_{\text{inj}}V_{\text{inj}}}\{{\left[S\right]}_{i}–{\left[S\right]}_{i‐1}+\frac{V_{\text{inj}}}{V_{\text{cell}}}(\frac{{\left[S\right]}_{i}+{\left[S\right]}_{i‐1}}{2})\}$$ (1)

In Eq. 1, ∆*H_s_*° is the standard enthalpy change accompanying the formation of complex *S*; *V*_cell_ is the volume of the reaction cell; [*R*]_inj_ is molar concentration of the reactant in the injection syringe; *V*_inj_ is the injection volume; and [*S*]_i_ and [*S*]_i-1_ are the equilibrium molar concentrations of the complex in the reaction cell after *i*th and *i-1*th injections, respectively. In a system involving multiple equilibria, these concentrations are implicit functions of the equilibrium constants and total reactant concentrations, as shown in the following sections, and the sum of *q_p,i_* over all types of complexes formed corresponds to the total binding heat at the *i*th injection. A set of thermodynamic parameters yielding the sum of *q_p,i_* that best fits the observed binding heat was obtained by the nonlinear least squares minimization method in Igor Pro 5.03 (WaveMatrics) using the Levenberg-Marquardt algorithm.

In global fitting, fundamental parameters of the interaction models (e.g., binding constants and enthalpies) are globally applied to all datasets, whereas “local” parameters—specific to individual experiment—are applied to each dataset. The primary local parameters are the protein activity term discussed above and the heat of sample dilution generated during injection of a protein into the ITC reaction cell. The errors reported in Table 1 are SDs obtained from the global fits, pertaining to the uncertainties in both the model accuracy and measurement precision (i.e., model and experimental errors).

#### Monomer-dimer equilibrium of Nup53

The monomer-dimer equilibrium of 53core (denoted by A) is represented by the equilibrium constant *K*_sc_

$$2A↔A_{2}\;K_{\text{sc}}=\frac{\left[A_{2}\right]}{{\left[A\right]}^{2}}$$ (2)

The total concentration of protein (A) in the reaction cell or in the injection syringe is given by

$${\left[A\right]}_{\text{total}}=[A]+2[A_{2}]=[A]+2K_{\text{sc}}{\left[A\right]}^{2}$$ (3)

So, [A] is given by

$$[A]=\{‐1+{\left(1+8K_{\text{sc}}{\left[A\right]}_{\text{total}}\right)}^{0.5}\}/4K_{\text{sc}}$$ (4)

To determine *K*_sc_, a concentrated 53core solution was injected in 2-μl increments into the reaction cell (202.8 μl) containing buffer only. The heat of dimer dissociation into monomers was monitored, and the extent of the dissociation (≡λ) was calculated by

$$\lambda ={\left[A_{2}\right]}_{\text{ini}}–[A_{2}]$$ (5)

where [A_2_]_ini_ and [A_2_] are concentrations of the dimer before and after dissociation, respectively. These quantities can be calculated from [A]_total_ in the injection syringe and in the reaction cell using Eq. 4. In the fitting routine, λ is substituted for the general concentration term [S] in Eq. 1.

#### Interactions between Nup53 and Kap121

In this section, 53core and Kap121 are denoted as A and B, respectively. In addition to the Nup53 dimerization equilibrium described above (Eq. 2), the equilibria describing interactions between Kap121 (B) and the dimeric (A_2_) or monomeric (A) states of 53core, with the binding stoichiometries explicitly considered, are given by

$$A_{2}+B↔A_{2}B\;K_{1}=\frac{\left[A_{2}B\right]}{\left[A_{2}][B\right]}$$ (6)

$$A_{2}+2B↔A_{2}B_{2}\;K_{2}=\frac{\left[A_{2}B_{2}\right]}{[A_{2}]{\left[B\right]}^{2}}$$ (7)

$$A+B↔\text{AB}\;K_{3}=\frac{\left[\text{AB}\right]}{\left[A][B\right]}$$ (8)

$$2A+B↔A_{2}*B\;K_{4}=\frac{\left[A_{2}*B\right]}{{\left[A\right]}^{2}[B]}$$ (9)

Complexes A_2_B and A_2_*B differ from each other in the oligomeric state of A (dimer versus two monomers; see Fig. 2F for a schematic representation of these complexes). The mass balance equations for the total concentrations of A and B are given by

$$\begin{array}{c} {\left[A\right]}_{\text{total}}=[A]+[\text{AB}]+2([A_{2}]+[A_{2}B]+[A_{2}B_{2}]+[A_{2}*B])=[A]+\\ K_{3}[A][B]+2K_{\text{sc}}{\left[A\right]}^{2}(1+K_{1}[B]+K_{2}{\left[B\right]}^{2})+2K_{4}{\left[A\right]}^{2}[B] \end{array}$$ (10)

$$\begin{array}{c} {\left[B\right]}_{\text{total}}=[B]+[\text{AB}]+[A_{2}B]+[A_{2}*B]+2[A_{2}B_{2}]\;=[B]+\\ K_{3}[A][B]+K_{1}K_{\text{sc}}{\left[A\right]}^{2}[B]+K_{4}{\left[A\right]}^{2}[B]+2K_{2}K_{\text{sc}}{\left[A\right]}^{2}{\left[B\right]}^{2} \end{array}$$ (11)

Equation 10 can be rearranged to solve a quadratic equation for [A]

$$[A]=\frac{-q+\sqrt{q^{2}-4\mathrm{pr}}}{2p}$$ (12)

where *p* = 2*K*_sc_(1 + *K*_1_[B] + *K*_2_[B]^2^) + 2*K*_4_[B], *q* = 1 + *K*_3_[B], and *r* = − [A]_total_.

Then, Eq. 11 can be numerically solved for [B] after replacing [A] by Eq. 12. The extent of the shift (≡λ) in the equilibrium between A and A_2_ by binding of B needs to be considered

$$\lambda =[A_{2}]‐{\left[A_{2}\right]}_{\text{ini}}+[A_{2}B]+[A_{2}B_{2}]$$ (13)

where [A_2_]_ini_ is the initial concentration of A_2_ in the reaction cell and can be calculated as described in the previous section (Eq. 4). In the fitting routine, [A_2_B], [A_2_B_2_], [AB], [A_2_*B], and λ are substituted for the general concentration term [S] in Eq. 1. This equilibrium scheme reduces to the one describing the interactions between the C-terminal IDR of Nup53 and Kap121 when *K*_sc_ = *K*_1_ = *K*_2_ = 0 (i.e., no contribution from the dimeric forms of Nup53).

The 53C- and 53core-Kap121 binding isotherms are clearly distinct, particularly in the first phase of the titration where the endothermic binding signal of the 2:1 binding mode is substantially increased for 53core (Fig. 1F). Because the RRM domain does not directly interact with Kap121 (Fig. 1G) and dimerizes in solution (Fig. 2C) and in the crystal structure (Fig. 2D), we reasoned that dimerization enhances the bivalent interactions between 53core and Kap121 (i.e., makes the 2:1 binding cooperative). Therefore, we introduced the binding constants and enthalpies for the interactions between dimeric 53core and Kap as fitting parameters, while these parameters for the monomeric 53core-Kap121 binding were fixed at values obtained for 53C-Kap121 titrations. However, it is also possible that the binding affinity of monomeric 53core for Kap121 is enhanced as compared to that of 53C due to the mere presence of the RRM domain.

To test this possibility, we performed global analysis of the 53core-Kap121 titrations in which we systematically increased the binding constants of the 1:1 and 2:1 monomeric 53core complexes with Kap121 as compared to those obtained for 53C-Kap121 interactions and monitored the fitting quality both visually (by comparing experimental data with the fitted curves) and statistically (χ^2^ value). As the Kap121 binding constants for monomeric 53core increase, the fitted curve progressively deviates from the titration data (fig. S4, A and B) and the χ^2^ value goes up (fig. S4C) The deviation is particularly pronounced for the exothermic second phase of the forward titration (or the first phase of the reverse titration), indicating that an increase in the affinity for 1:1 binding, which should originate from the mere presence of the RRM domain rather than from dimerization, is incompatible with experimental data. Overall, our systematic analysis justifies the use of the 53C-Kap binding constants in the global fitting of 53core-Kap titration data.

To further illuminate the critical role of the RRM domain dimerization, we fit the 53core-Kap121 titration data with a model in which the Kap121 binding affinity of the C-terminal IDR of Nup53 is enhanced because of the presence of the RRM domain without any additional contributions from RRM dimerization. In this model, the binding constants for the 53core_dimer_:Kap121 and 53core_dimer_:(Kap121)_2_ complexes are dictated by the 1:1 binding constant of monomeric Nup53 (i.e., 2*K* and *K*^2^). The χ^2^ value obtained from this model is almost twofold greater than that from our original model. The deviation from experimental data is more pronounced for the forward titrations performed at high Nup53 concentrations (fig. S4D). Therefore, the dimerization-induced increase in the 2:1 binding constant needs to be included in the analysis to satisfyingly fit the endothermic phase enhanced at high Nup53 concentrations.

#### Interactions between Nup53 and Nic96 in the presence of Kap121

ITC data obtained for titrating Nic96 into the mixture containing 53core (A) and Kap121 (B) were initially analyzed by a single-site 1:1 model to obtain the apparent binding constant for the Nic96-53core interactions. This value was compared with the binding constant obtained in the absence of Kap121 (≡ ***K***_**5**_) to estimate the overall allosteric effect of Kap121 binding to the C-terminal IDR of 53core on the interaction between Nic96 and the N-terminal IDR of 53core. To distinguish between the allosteric effects arising from monomeric and dimeric states of 53core, we analyzed all equilibria for the ternary system including 53core dimerization as well as 53core-Kap121, Nic96-53core, and Nic96-53core-Kap121 interactions. In this analysis, three types of binding constants were defined: (i) ***K***_**5**_ describing Nic96 interactions with the N-IDR of monomeric or dimeric 53core—all containing free C-terminal IDR (i.e., without the bound Kap; A and A_2_); (ii) ***K***_**6**_ describing Nic96 interactions with the N-IDR of dimeric 53core containing the C-terminal IDR occupied by Kap121 (A_2_B and A_2_B_2_); and (iii) ***K***_**7**_ describing Nic96 interactions with the N-IDR of monomeric 53core whose C-terminal IDR is occupied by Kap121 (AB and A_2_*B). For any reactants with the two copies of A (A_2_, A_2_B, A_2_B_2_, and A_2_*B), these “site N-IDR” binding constants have to be multiplied or squared by a factor of 2 when the reactants bind one or two molecules of Nic96, respectively. Then, the mass balance equations for the total concentrations of A, B, and C are given by

$$\begin{array}{c} {\left[A\right]}_{\text{total}}=[A](1+K_{5}[C])+2[A_{2}]{\left(1+K_{5}[C]\right)}^{2}+\\ 2([A_{2}B]+[A_{2}B_{2}]){\left(1+K_{6}[C]\right)}^{2}+\\ {}[\text{AB}](1+K_{7}[C])+2[A_{2}*B]{\left(1+K_{7}[C]\right)}^{2}\\ =[A](1+K_{5}[C])+2\;K_{\text{sc}}{\left[A\right]}^{2}{\left(1+K_{5}[C]\right)}^{2}+\\ 2\;K_{\text{sc}}{\left[A\right]}^{2}(K_{1}[B]+K_{2}{\left[B\right]}^{2}){\left(1+K_{6}[C]\right)}^{2}+\\ K_{3}[A][B](1+K_{7}[C])+2\;K_{4}{\left[A\right]}^{2}[B][(1+K_{7}[C]){}^{2} \end{array}$$ (14)

$$\begin{array}{c} {\left[B\right]}_{\text{total}}=[B]+[\text{AB}](1+K_{7}[C])+[A_{2}*B]{\left(1+K_{7}[C]\right)}^{2}+\\ ([A_{2}B]+2[A_{2}B_{2}]){\left(1+K_{6}[C]\right)}^{2}\\ =[B]+K_{3}[A][B](1+K_{7}[C])+K_{4}{\left[A\right]}^{2}[B]{\left(1+K_{7}[C]\right)}^{2}+\\ K_{\text{sc}}{\left[A\right]}^{2}(K_{1}[B]+2\;K_{2}{\left[B\right]}^{2}){\left(1+K_{6}[C]\right)}^{2} \end{array}$$ (15)

$$\begin{array}{c} {\left[C\right]}_{\text{total}}=[C]+[\text{AC}]+[A_{2}C]+2[A_{2}C_{2}]+\\ {}[\text{ABC}]+[A_{2}\text{BC}]+2[A_{2}{\text{BC}}_{2}]+[A_{2}B_{2}C]+\\ 2[A_{2}B_{2}C_{2}]+[A_{2}*\text{BC}]+2[A_{2}*{\text{BC}}_{2}]\\ =[C]+K_{5}[A][C]+2\;K_{5}K_{\text{sc}}{\left[A\right]}^{2}[C]+\\ 2\;{K_{5}}^{2}K_{\text{sc}}{\left[A\right]}^{2}{\left[C\right]}^{2}+K_{7}K_{3}[A][B][C]+\\ 2\;K_{6}K_{1}K_{\text{sc}}{\left[A\right]}^{2}[B][C]+2\;{K_{6}}^{2}K_{1}K_{\text{sc}}{\left[A\right]}^{2}[B]{\left[C\right]}^{2}+\\ 2\;K_{6}K_{2}K_{\text{sc}}{\left[A\right]}^{2}{\left[B\right]}^{2}[C]+2\;{K_{6}}^{2}K_{2}K_{\text{sc}}{\left[A\right]}^{2}{\left[B\right]}^{2}{\left[C\right]}^{2}+\\ 2\;K_{7}K_{4}{\left[A\right]}^{2}[B][C]+2\;{K_{7}}^{2}K_{4}{\left[A\right]}^{2}[B]{\left[C\right]}^{2} \end{array}$$ (16)

Equation 14 can be rearranged to solve a quadratic equation for [A]

$$[A]=\frac{-q+\sqrt{q^{2}-4\mathrm{pr}}}{2p}$$ (17)

where *p* = 2*K*_sc_(1 + *K*_5_[C])^2^ + 2(*K*_1_*K*_sc_[B] + *K*_2_*K*_sc_[B]^2^)(1 + *K*_6_[C])^2^ + 2 *K*_4_[B](1 + *K*_7_[C])^2^, *q* = 1 + *K*_5_[C] + *K*_3_[B](1 + *K*_7_[C]), and *r* = − [A]_total_. Then, Eqs. 15 and 16 can be simultaneously solved for [B] and [C], respectively, after replacing [A] by Eq. 17. The extent of the shift (λ) in the oligomerization equilibrium of A and in the binding equilibria of A and B needs to be considered

$$\lambda_{A2}=[A_{2}]+[A_{2}C]+[A_{2}C_{2}]+\lambda_{A2B}+\lambda_{A2B2}–{\left[A_{2}\right]}_{\text{ini}}$$ (18)

$$\lambda_{A2B}=[A_{2}B]+[A_{2}\text{BC}]+[A_{2}{\text{BC}}_{2}]–{\left[A_{2}B\right]}_{\text{ini}}$$ (19)

$$\;\lambda_{A2B2}=[A_{2}B_{2}]+[A_{2}B_{2}C]+[A_{2}B_{2}C_{2}]–{\left[A_{2}B_{2}\right]}_{\text{ini}}$$ (20)

$$\lambda_{\text{AB}}=[\text{AB}]+[\text{ABC}]–{\left[\text{AB}\right]}_{\text{ini}}$$ (21)

$$\;\lambda_{A2}{*}_{B}=[A_{2}*B]+[A_{2}*\text{BC}]+[A_{2}*{\text{BC}}_{2}]–{\left[A_{2}*B\right]}_{\text{ini}}$$ (22)

where [A_2_]_ini_, [A_2_B]_ini_, [A_2_B_2_]_ini_, [AB]_ini_, and [A_2_*B]_ini_ are the initial concentrations of the distinct molecular states of A and AB in the reaction cell and can be calculated as described above. In the fitting routine, [AC], [A_2_C], [A_2_C_2_], [ABC], [A_2_BC], [A_2_BC_2_], [A_2_B_2_C], [A_2_B_2_C_2_], [A_2_*BC], [A_2_*BC_2_], λ_A2_, λ_A2B_, λ_A2B2_, λ_AB_, and λ_A2_*_B_ are substituted for the general concentration term [S] in Eq. 1. The value of *K*_5_ describing Nic96 binding to 53core was determined using the single-site 1:1 binding model. This equilibrium scheme can be reduced to the one describing the interactions between the monomeric 53core and Nic96 in the presence of Kap121 when *K*_sc_ = *K*_1_ = *K*_2_ = *K*_6_ = 0. Using this reduced scheme, the data for the titrations of monomeric Nup53 with Nic96 in the presence of Kap121 were first analyzed to obtain the value of *K*_7_. The values of *K*_5_ and *K*_7_ were then used in the subsequent analysis of Nic96-53core titrations in the presence of Kap121 to obtain the value of *K*_6_.

### Crystallization and structure determination of the yeast Nup53 RRM domain

Crystals of the Nup53 RRM domain from *S. cerevisiae* were grown at 4°C in sitting drops containing 1 μl of the protein (at 5 mg/ml) and 1 μl of a reservoir solution consisting of 20% (w/v) PEG-3350 (polyethylene glycol, molecular weight 800) and 0.2 M ammonium sulfate. The crystals belong to the space group *C*222_1_ and contain two RRM molecules in the asymmetric unit. For cryoprotection, crystals were stabilized in the mother liquor supplemented with 15% (v/v) glycerol and flash-frozen in liquid nitrogen. X-ray diffraction data were collected at the Advanced Light Source, Lawrence Berkeley National Laboratory, beamline 8.2.1. The data were integrated and scaled using the HKL2000 package (*49*). The structure of the Nup53 homodimer was solved by molecular replacement (MR) using MOLREP (*50*) from the CCP4 (Collaborative Computational Project) program suite with the Nup53 RRM structure from *Pichia guilliermondii* [Protein Data Bank (PDB) accession code: 3P3D] as a search model. After MR, iterative cycles of manual rebuilding were carried out in COOT (*51*), and the final model was refined at a resolution of 1.75 Å using restrained refinement in PHENIX (*52*). The Fitmunk server (*53*) was used to identify improved side-chain rotamers. No electron density was observed for residues 287 to 304 due to predicted disorder. The stereochemical quality of the structural model was assessed with MolProbity (*54*). There were no residues in the disallowed region of the Ramachandran plot. Details of the data collection, phasing, and refinement statistics are provided in table S2. Structure-guided sequence alignment was generated with BioEdit (www.mbio.ncsu.edu/BioEdit/bioedit.html). All figures containing the structure of the Nup53 RRM domain were generated with PYMOL (https://pymol.org/2/).

### Negative-stain EM

Negative staining was carried out using protein complexes deposited on glow-discharged, 200-square-mesh carbon-coated copper grids (Electron Microscopy Sciences). Gel filtration–purified samples containing Kap121 or its complex with 53C, 53core, or 53core and Nic96 (all mixed at equimolar ratios) were diluted up to 10-fold before they were stained with 2% (w/v) uranyl acetate and analyzed. Micrographs were collected on the JEOL 1400 Plus Transmission Electron Microscope operating at 120 kV, with a Gatan 2K × 2K digital camera at 40,000× nominal magnification (2.5 Å/pixel) and a defocus range of −0.8 to −1.5 μm. Particle picking and image analysis were performed using RELION v2.1 (*55*). A small subset of particles was initially picked manually to generate 2D class averages by reference-free classification. These 2D classes were subsequently low pass–filtered to a resolution of 50 Å and used as references for automated particle picking. Particles that were picked incorrectly or were not well centered in the class averages were removed from subsequent stages of the analysis. A final set of autopicked particles was subjected to three rounds of 2D classification and grouped into 50 to 100 classes, which were sufficient to represent different particle orientations. For optimal 3D classification, these particles were first subjected to 3D refinement using the crystal structure of Kap121 (PDB code: 3W3T), low pass–filtered to 60 Å, as a reference. The map from a 3D refinement was then used in 3D classification, which was carried out by setting the regularization parameter *T* to 4 and gradually fine-tuning the image alignment sampling to a final setting of 35 iterations with an angular sampling interval of 7.5°, offset search range set to 5 pixels, and step to 1 pixel. The final refined 3D maps were calculated using 17,014 particles for Kap121, 30,941 for the Kap121 complex with 53C, 54,055 for 53core·Kap121 complexes, and 16,055 for the 2:2 ternary complex containing Kap121, 53core, and Nic96. The quality of the obtained maps was sufficient to distinguish various conformational states of the Kap121-containing complexes from the unbound/dissociated Kap121 particles present, to varying degrees, in all tested samples. To visualize in detail the stem structure linking two Kap121 molecules within the 2:2 assembly of the 53core·Kap121 and Kap121·53core·Nic96 complexes, additional particle alignment was performed with a binary mask encompassing exclusively this region. Crystal structures of Kap121 (PDB code: 3W3T), Nic96 (PDB code: 2QX5), and the Nup53 RRM domain (PDB code: 5UAZ) were fitted into the final density maps with high cross-correlation scores (between 0.8 and 1.0) in UCSF (University of California, San Francisco) Chimera (*56*). However, the unique orientation of the RRM domain within the stem region of the 53core·Kap121 complex could not be unambiguously determined at the resolution of the NS-EM map.

### Limited proteolysis

Limited proteolysis experiments were performed in 20 mM Hepes (pH 7.5), 200 mM NaCl, and 3 mM DTT at ambient room temperature. Trypsin was added to ~5 μg of the 53C fragment alone or to its 1:1 molar complex with Kap121 at an enzyme/protein ratio of 1:25 (w/w). The total reaction mixture volume for each sample was 15 μl. The digestions were stopped at specified time intervals (15, 30, 45, 60, 90, and 120 min) by the addition of the SDS sample buffer. The reaction products (10 μl) were heated immediately at 95°C for 5 min and separated by SDS-PAGE on a 4 to 12% gradient gel and visualized by Coomassie brilliant blue staining.

### Multi-angle light scattering

Purified human Nup35 RRM domain was concentrated to 15 mg/ml and injected into a size exclusion chromatography column (Superdex 200 10/300 GL column, GE Healthcare) connected to multiangle light scattering and refractive index detectors (DAWN HELEOS and Optilab rEX; Wyatt Technology). The analysis was performed in the ITC binding buffer at ambient room temperature. Weight-averaged molar masses were determined by multiangle light scattering as previously described (*33*) using the ASTRA 6.0.2.9 software (Wyatt Technology).

### Circular dichroism (CD) spectroscopy

Samples for CD spectroscopy were dialyzed against 10 mM HK_2_PO_4_ buffer (pH 7.5) supplemented with 150 mM NaCl, filtered, and centrifuged before the experiments. CD spectra for the 53NTD and 53C fragments (each at 10 μM) were recorded from 320 to 195 nm at room temperature, using an Aviv model 62 DS CD spectrometer with a quartz cuvette (path length = 0.1 cm). For each sample, the measurement was performed twice to confirm that the sample was completely equilibrated at a given temperature and that there was no time dependence in the CD spectra. The spectra were corrected for an instrumental offset and the signal from a buffer, by subtracting the CD signal and the control spectra of a blank buffer from the recorded spectra, respectively. The corrected CD spectra were converted to mean residue molar ellipticity (θ) (*57*). The Nup53NTD and Nup53C spectra were analyzed and plotted by CAPITO (*58*).

## Supplementary Material

http://advances.sciencemag.org/cgi/content/full/5/11/eaax1836/DC1

Download PDF

Allosteric modulation of nucleoporin asemblies by intrinsically disordered regions

## SUPPLEMENTARY MATERIALS

Supplementary material for this article is available at http://advances.sciencemag.org/cgi/content/full/5/11/eaax1836/DC1 Fig. S1. Characterization of the N- and C-terminal regions of Nup53 and their interactions with Nic96, Nup157, and Kap121. Fig. S2. Kap121 interacts with the C-terminal region of Nup53 using the NLS and FG motif binding sites. Fig. S3. The RRM domain of human Nup53 forms a constitutive dimer in solution. Fig. S4. The RRM domain enhances biphasic profile of 53core-Kap121 interactions. Fig. S5. NS-EM analysis of Nup53-Kap121 interactions. Fig. S6. Reconstitution of Nup53 into complexes with Nic96 and Nup157. Fig. S7. Kap121 allosterically destabilizes Nic96 interactions with dimerization-deficient Nup53. Fig. S8. NS-EM analysis of the Kap121·53core·Nic96 complex. Table S1. Bacterial expression constructs. Table S2. Data collection and refinement statistics for the *Sc*Nup53 RRM domain (molecular replacement). References (*58*–*61*)

## Appendix

View/request a protocol for this paper from *Bio-protocol*.
