# Supplementary material for: Allosteric modulation of nucleoporin assemblies by intrinsically disordered regions
Source: Sci Adv. 2019 Nov 27;5(11):eaax1836. doi: 10.1126/sciadv.aax1836 (PMC6881172; doi:10.1126/sciadv.aax1836)
Supplement: http://advances.sciencemag.org/cgi/content/full/5/11/eaax1836/DC1 [file supp_5_11_eaax1836__index.html]

Science Advances | Science AdvancesAAASSearchScience AdvancesMenu

## Supplementary Materials

**This PDF file includes:**

- Fig. S1. Characterization of the N- and C-terminal regions of Nup53 and their interactions with Nic96, Nup157, and Kap121.
- Fig. S2. Kap121 interacts with the C-terminal region of Nup53 using the NLS and FG motif binding sites.
- Fig. S3. The RRM domain of human Nup53 forms a constitutive dimer in solution.
- Fig. S4. The RRM domain enhances biphasic profile of 53core-Kap121 interactions.
- Fig. S5. NS-EM analysis of Nup53-Kap121 interactions.
- Fig. S6. Reconstitution of Nup53 into complexes with Nic96 and Nup157.
- Fig. S7. Kap121 allosterically destabilizes Nic96 interactions with dimerization-deficient Nup53.
- Fig. S8. NS-EM analysis of the Kap121·53core·Nic96 complex.
- Table S1. Bacterial expression constructs.
- Table S2. Data collection and refinement statistics for the *Sc*Nup53 RRM domain (molecular replacement).
- References (*58*–*61*)

Download PDF

**Files in this Data Supplement:**

- Adobe PDF - aax1836\_SM.pdf
