# Supplementary material for: Allosteric modulation of nucleoporin assemblies by intrinsically disordered regions
Source: Sci Adv. 2019 Nov 27;5(11):eaax1836. doi: 10.1126/sciadv.aax1836 (PMC6881172; doi:10.1126/sciadv.aax1836)
Supplement: Download PDF [file aax1836_SM.pdf]

Science Advances
AAAS

advances.sciencemag.org/cgi/content/full/5/11/eaax1836/DC1

Supplementary Materials for

Allosteric modulation of nucleoporin assemblies by intrinsically disordered regions

Bartlomiej Jan Blus*, Junseock Koh, Aleksandra Krolak, Hyuk-Soo Seo, Elias Coutavas, Günter Blobel

*Corresponding author. Email: bblus@rockefeller.edu

Published 27 November 2019, *Sci. Adv.* **5**, eaax1836 (2019)
DOI: 10.1126/sciadv.aax1836

This PDF file includes:

Fig. S1. Characterization of the N- and C-terminal regions of Nup53 and their interactions with Nic96, Nup157, and Kap121.
Fig. S2. Kap121 interacts with the C-terminal region of Nup53 using the NLS and FG motif binding sites.
Fig. S3. The RRM domain of human Nup53 forms a constitutive dimer in solution.
Fig. S4. The RRM domain enhances biphasic profile of 53core-Kap121 interactions.
Fig. S5. NS-EM analysis of Nup53-Kap121 interactions.
Fig. S6. Reconstitution of Nup53 into complexes with Nic96 and Nup157.
Fig. S7. Kap121 allosterically destabilizes Nic96 interactions with dimerization-deficient Nup53.
Fig. S8. NS-EM analysis of the Kap121·53core·Nic96 complex.
Table S1. Bacterial expression constructs.
Table S2. Data collection and refinement statistics for the *Sc*Nup53 RRM domain (molecular replacement).
References (*58–61*)

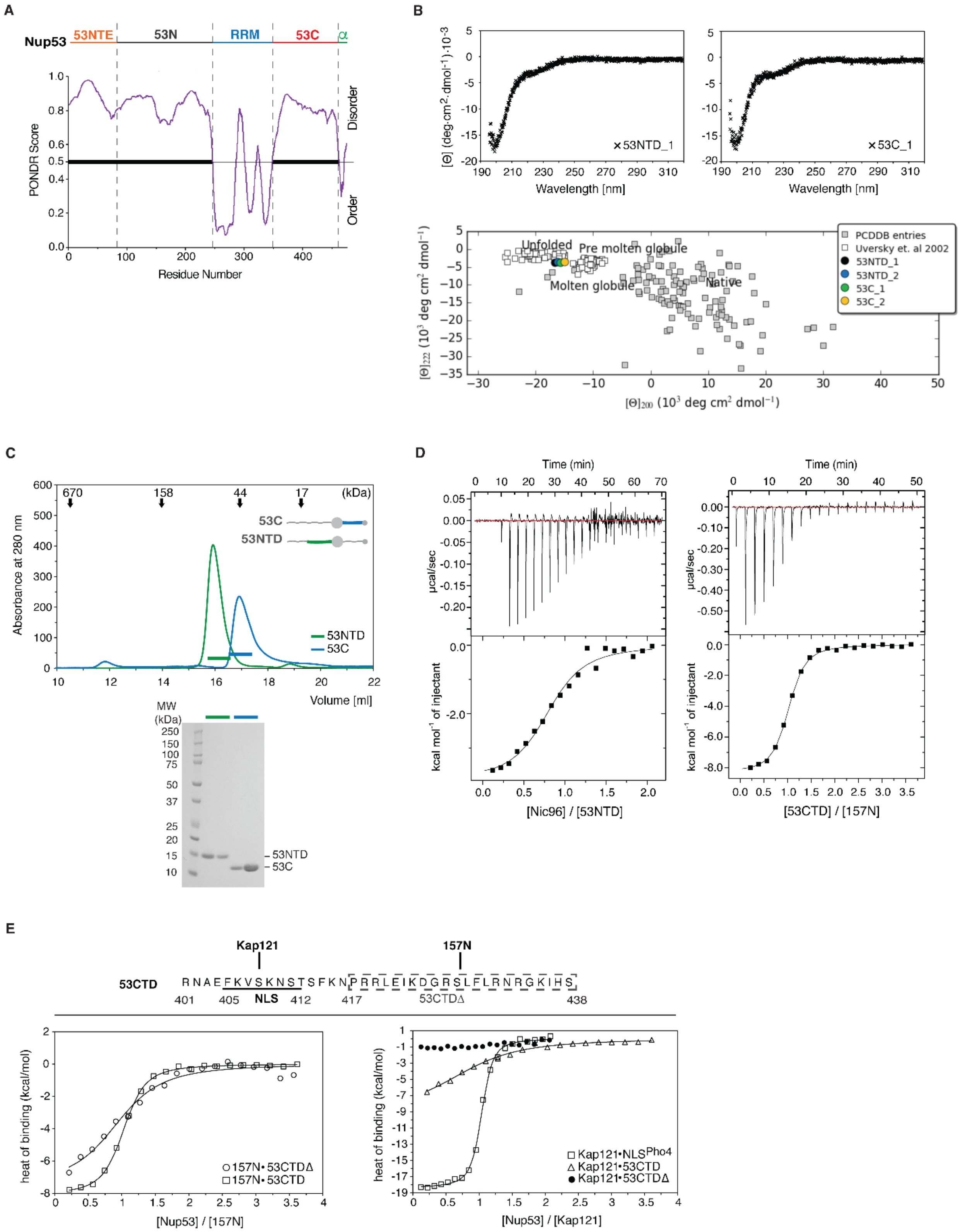
A
Nup53
53NTE
53N
RRM
53C
α
PONDR Score
Residue Number
Disorder
Order
B
[Θ] (deg·cm2·dmol-1)·10-3
Wavelength [nm]
×53NTD_1
×53C_1
Unfolded
Pre molten globule
Molten globule
Native
PCDDB entries
Uversky et. al 2002
53NTD_1
53NTD_2
53C_1
53C_2
[Θ]222 (10³ deg cm² dmol⁻¹)
[Θ]200 (10³ deg cm² dmol⁻¹)
C
Absorbance at 280 nm
Volume [ml]
670
158
44
17
(kDa)
53C
53NTD
MW (kDa)
250
150
100
75
50
37
25
20
15
10
D
Time (min)
µcal/sec
kcal mol⁻¹ of injectant
[Nic96] / [53NTD]
[53CTD] / [157N]
E
Kap121
157N
53CTD
R N A E F K V S K N S T S F K N P R R L E I K D G R S L F L R N R G K I H S
401
405
NLS
412
417
53CTDΔ
438
heat of binding (kcal/mol)
157N•53CTDΔ
157N•53CTD
[Nup53] / [157N]
Kap121•NLSPho4
Kap121•53CTD
Kap121•53CTDΔ
[Nup53] / [Kap121]

Fig. S1. Characterization of the N- and C-terminal regions of Nup53 and their interactions with Nic96, Nup157, and Kap121. (**A**) Disorder prediction results for full-length Nup53 were obtained from the Predictor of Natural Disordered Regions (PONDR) web server (www.pondr.com). Distinct regions of Nup53 are highlighted above the prediction plot (**B**) Far-UV circular dichroism (CD) spectra of the 53NTD and 53C fragments are shown (top). For each fragment, the spectra were collected in duplicate (e.g. 53NTD_1 and 53NTD_2) and analyzed by the CAPITO web server (*58*) (see Material and Methods for details). The spectra were compared to the Protein Circular Dichroism Data Bank (PCDDB) dataset containing a large number of folded proteins (*59*) and to the reference containing 95 CD spectra for unfolded and pre-molten globule states (*60*) (bottom). (**C**) A composite chromatogram showing size-exclusion chromatography (SEC) elution profiles for 53NTD (green, 250 μM) and 53C (blue, 750 μM). Below, peak elution fractions for each Nup53 fragment were resolved by SDS-PAGE. Calculated molecular weights are as follows: 53NTD (10.3 kDa) and 53C (9.3 kDa). Based on these elution profiles, the estimated hydrodynamic radii for 53NTD and 53C are 32.4 Å and 27.0 Å, respectively, which are comparable to the predicated values for extended, disordered conformations (53NTD-27.9 Å, 53C-26.4 Å) (*61*). Elution volumes for the protein molecular weight standards are shown above the chromatogram (thyroglobulin: 670 kDa, γ-globulin: 158 kDa, ovalbumin: 44 kDa, myoglobin: 17 kDa). (**D**) Raw ITC heat signals generated in titration of 53NTD (20 μM) or 157N (25 μM) with sequential injections of Nic96 (250 μM) or 53CTD (400 μM) are shown in top panel as time traces. Integrated heat signals were analyzed by a nonlinear regression method with a single-site 1:1 binding model (continuous lines; bottom panel). (**E**) ITC binding isotherms obtained for titrations of (left panel) 157N (25 μM) with 53CTD (400 μM) or 53CTDΔ (375 μM) and (right panel) Kap121 (25 μM) with 53CTD (400 μM) or 53CTDΔ (250 μM) or titration of Kap121 (15 μM) with the NLS peptide (150 μM) derived from the yeast Pho4 transcription factor (residues 140-166; Uniprot accession code: P07270). The 53CTD and 53CTDΔ sequences (residues 401-438 and 417-438, respectively) are shown above the titrations. The underlined portion of 53CTD indicates the NLS peptide region bound to Kap121 in the crystal structure (PDB accession code: 3W3Y). Note that 53CTD specifically interacts with either Kap121 or 157N (with the binding constants of 2.1 (±0.7) x 10^5 M^{-1} and 1.6 (± 0.2) x 10^6 M^{-1}, respectively), whereas the 53CTDΔ fragment lacking the NLS sequence is only selective for 157N. The NLS peptide in Pho4 binds Kap121 with a stoichiometry ratio of 1:1 and a binding constant of 9.2 (± 1.0) x 10^6 M^{-1}.

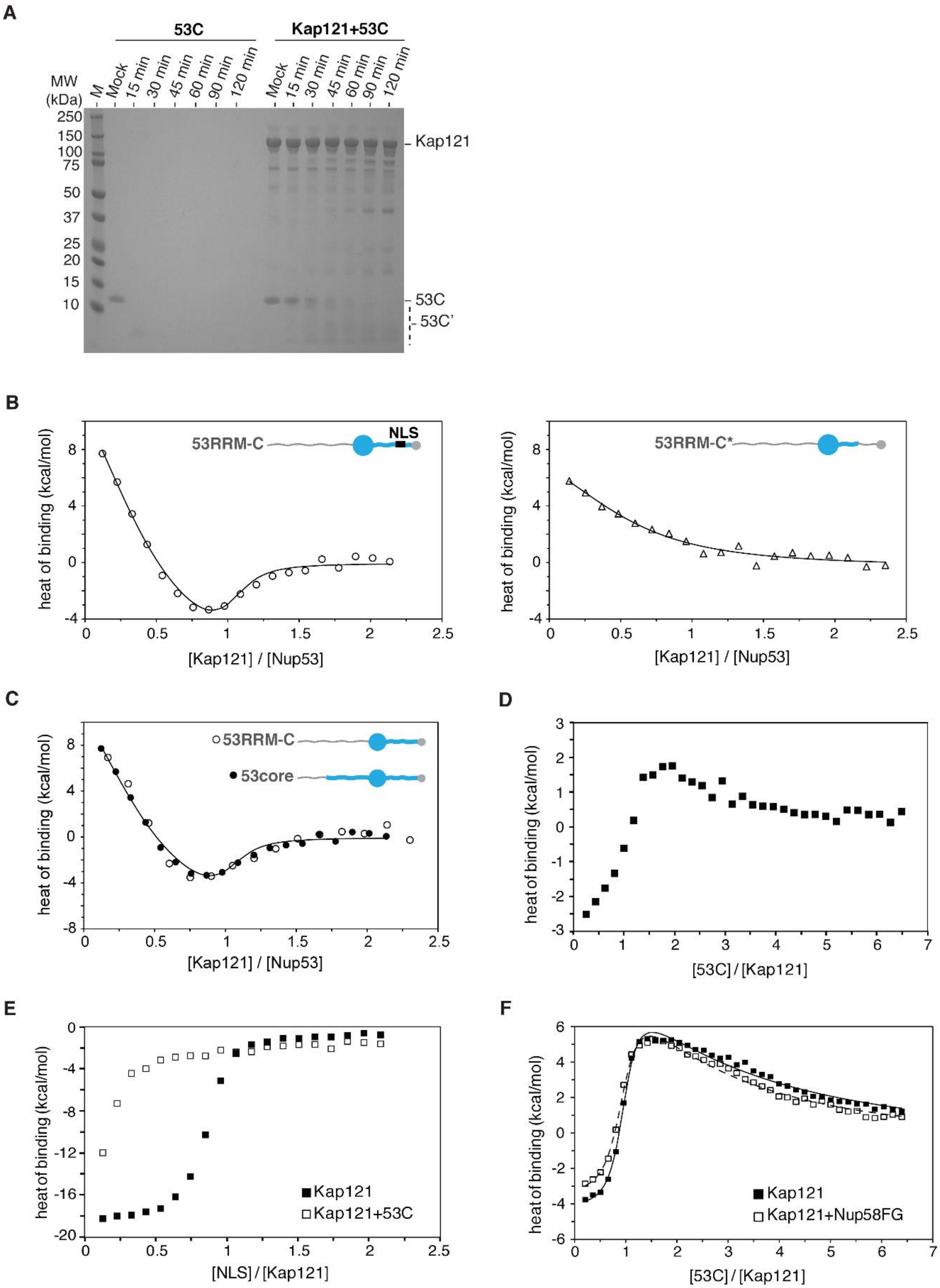
A
53C
Kap121+53C
MW (kDa)
M
Mock
15 min
30 min
45 min
60 min
90 min
120 min
250
150
100
75
50
37
25
20
15
10
Kap121
53C
53C'
B
53RRM-C
NLS
heat of binding (kcal/mol)
[Kap121] / [Nup53]
53RRM-C*
C
53RRM-C
53core
D
[53C] / [Kap121]
E
Kap121
Kap121+53C
[NLS] / [Kap121]
F
Kap121
Kap121+Nup58FG
[53C] / [Kap121]

Fig. S2. Kap121 interacts with the C-terminal region of Nup53 using the NLS and FG motif binding sites. (**A**) Probing the Kap121·53C complex structure by limited proteolysis. Protein samples containing ~5 μg of 53C alone or its stochiometric complex with Kap121 were mixed with trypsin at the 25:1 (wt/wt) ratio and incubated at ambient room temperature. The reactions were stopped by adding SDS sample buffer at indicated time points and analyzed by SDS-PAGE (M = molecular-weight marker). The 53C' bands represent the 53C cleavage products generated by trypsin digestion (see Materials and Methods for details). (**B**) Binding isotherms for interactions of Kap121 (150 μM) with the C-terminal truncation fragments of Nup53 (each at 15 μM): 53RRM-C (left panel) and 53RRM-C* (right panel), as monitored by ITC. 53-RRM-C* lacks the 53CTD region that contains the NLS sequence. (**C**) Binding isotherms for interactions of Kap121 (150 μM) with 53RRM-C (15 μM) and 53core (15 μM), as monitored by ITC. These binding isotherms are thermodynamically equivalent. (**D-F**) Binding isotherms obtained from titration of (D) Kap121 (20 μM) with 53C (510 μM), (E) Kap121 (15 μM) with the NLS peptide from the Pho4 protein in the absence or presence of 53C (15 μM), and (F) Kap121 (20 μM) with 53C (600 μM) in the absence or presence of the FG-rich region of *Rn*Nup58 (30 μM). The two reverse titrations in (F) were collected in a buffer with reduced salt concentration (75 mM NaCl) to enhance Nup58FG binding to Kap121. The thermograms were semi-quantitively fitted with a two-mode binding model used for the analysis of the 53C-Kap121 interactions in the ITC buffer containing 150 mM NaCl (**Fig. 2A**). The resulting parameters for the 53C-Kap121 titrations are $K = 2.1\ (\pm 0.3) \times 10^{6}\ M^{-1}$ and $\Delta H^{0} = -3.2\ (\pm 0.2)$ kcal/mol for the 1:1 binding mode and $K = 2.2\ (\pm 0.3) \times 10^{10}\ M^{-2}$ and $\Delta H^{0} = 43.5\ (\pm 1.7)$ kcal/mol for the 2:1 binding mode, respectively. In the presence of the Nup58FG fragment, thermodynamic parameters for the 1:1 binding mode are $K = 8.8\ (\pm 1.6) \times 10^{5}\ M^{-1}$ and $\Delta H^{0} = -2.4\ (\pm 0.1)$ kcal/mol; and $K = 7.2\ (\pm 1.4) \times 10^{9}\ M^{-2}$ and $\Delta H^{0} = 32.9\ (\pm 1.0)$ kcal/mol for the 2:1 binding mode.

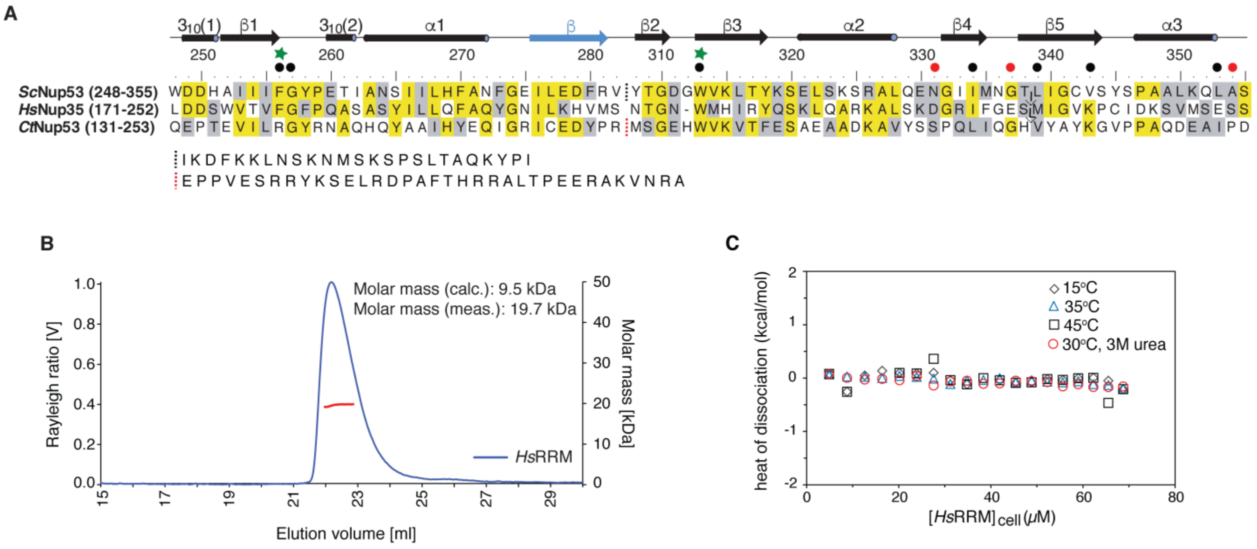

Fig. S3. The RRM domain of human Nup53 forms a constitutive dimer in solution. (**A**) A structure-guided sequence alignment of the selected Nup53 RRM homologs is shown together with the secondary structure elements above the alignment (β-strand in blue is unique to the human RRM domain). The identical and similar residues in the alignment are highlighted with yellow and gray boxes, respectively. The residues involved in the *Sc*RRM homodimerization interface are indicated with dots of different color describing a particular type of the interaction (black for hydrophobic and van der Waals interactions, red for a hydrogen bond). The green stars mark the conserved residues essential for the dimerization of yeast and human RRMs. Dotted black and red vertical lines represent the non-conserved insertion sequences in *S. cerevisiae* and *C. thermophilum* Nup53 (uniprot accession code: G0S156) RRMs. (**B**) A molar mass determination of the human RRM domain (from Nup35) by MALS coupled to SEC (sample injected at 15 mg/ml). The Rayleigh ratio (blue) and the molar mass (red) are shown as a function of the elution volume. The determined molar mass (19.7 kDa) is consistent with the formation of the Nup35 RRM dimer. (**C**) ITC dissociation profiles of the *Hs*RRM domain from Nup35 (all at 400 μM) were obtained at different temperatures in the absence or presence of 3M urea. No appreciable heat of dissociation was detected at any of the tested conditions, suggesting that the dimerization interface in the human Nup35 RRM forms much tighter contacts than the one in the yeast counterpart.

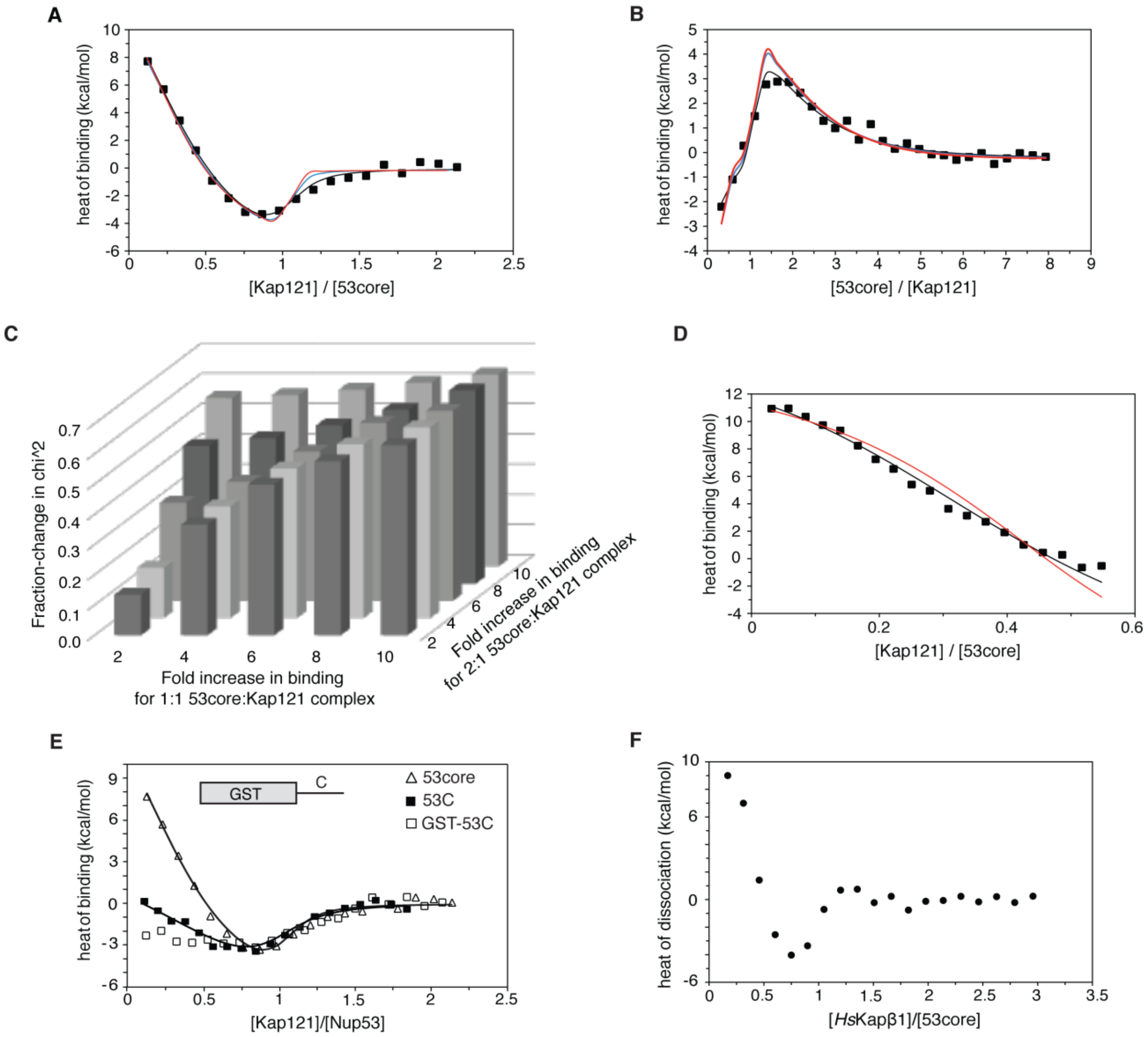

Fig. S4. The RRM domain enhances biphasic profile of 53core-Kap121 interactions. (**A-B**) Binding isotherms obtained from (A) forward titration of Kap121 (150 μM) into 53core (15 μM; reproduced from **Fig. 2E**) and from (B) reverse titration of 53core (430 μM) into Kap121 (17 μM), as monitored by ITC. These isotherms were globally analyzed together with those in **Fig. 2E** by a multi-equilibrium model (see Materials and Methods for details). Global fit (black lines) using the binding constants of the monomeric 53core-Kap121 interactions fixed at the K's of the 53C-Kap121 interactions yielded binding parameters for the dimeric 53core-Kap121 complexes **(Table 1).** Using the increased binding constants of monomeric 53core-Kap121 interactions—by 4-fold (blue lines) or 10-fold (red lines)—as compared to those of 53C-Kap121 interactions

results in pronounced deviations of the fitted binding curve from experimental data. (**C**) The χ^2 value, expressed as fractional increase between the alternative models and our original model, systematically increases with higher binding constants of monomeric 53core-Kap121 complexes, lowering data fit quality. (**D**) A binding isotherm for the forward titration of Kap121 (150 μM) into 53core (70 μM) (reproduced from **Fig. 2E**). Global fit with a model where the Kap121 binding affinity for the C-terminal IDRs of 53core is enhanced due to presence of the RRM domain, without contributions from its dimerization, resulted in a worse fit (red line) than our original model (black line), increasing the χ^2 value by 2-fold. (**E**) A titration of Kap121 (150 μM) into GST-53C (15 μM) is overlaid with 53core and 53C binding isotherms (both at 15 μM). (**F**) A representative binding isotherm obtained from titration of the human Kapβ1 fragment (77 μM) into 53core (5.4 μM), as monitored by ITC.

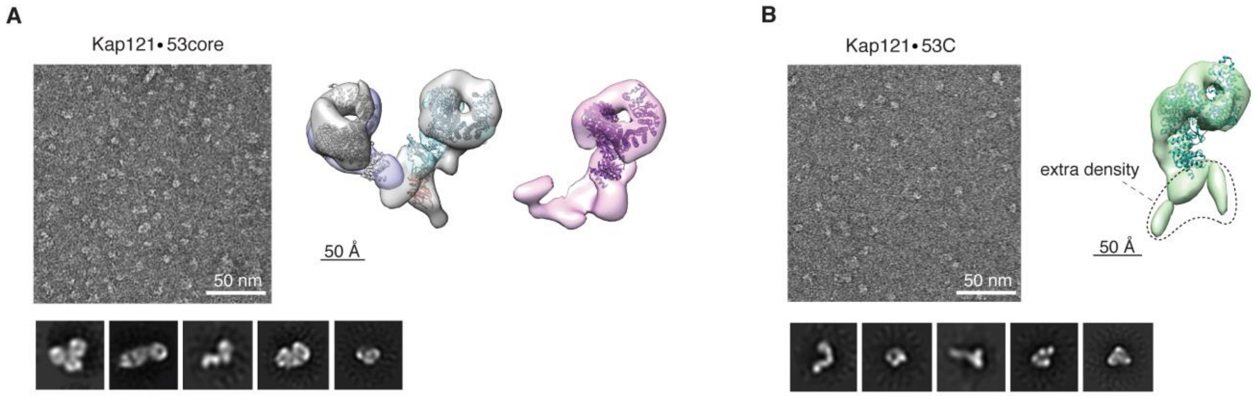

Fig. S5. NS-EM analysis of Nup53-Kap121 interactions. Negative-stain EM analysis of (**A**) Kap121·53core and (**B**) Kap121·53C complexes. A representative electron micrograph for each complex (top left panels) is shown with select 2D class averages (bottom panels). The 3D reconstructions are displayed with fitted crystal structures of Kap121 (PDB code: 3W3T; top right panels). The crystal structure of the Nup53 RRM domain (PDB code: 5UAZ; orange) is docked into the dimeric conformation of the Kap121·53core model (panel A). The density regions encircled with a dashed line in the Kap121·53C reconstitution presumably correspond to the bound 53C fragment(s) (panel B).

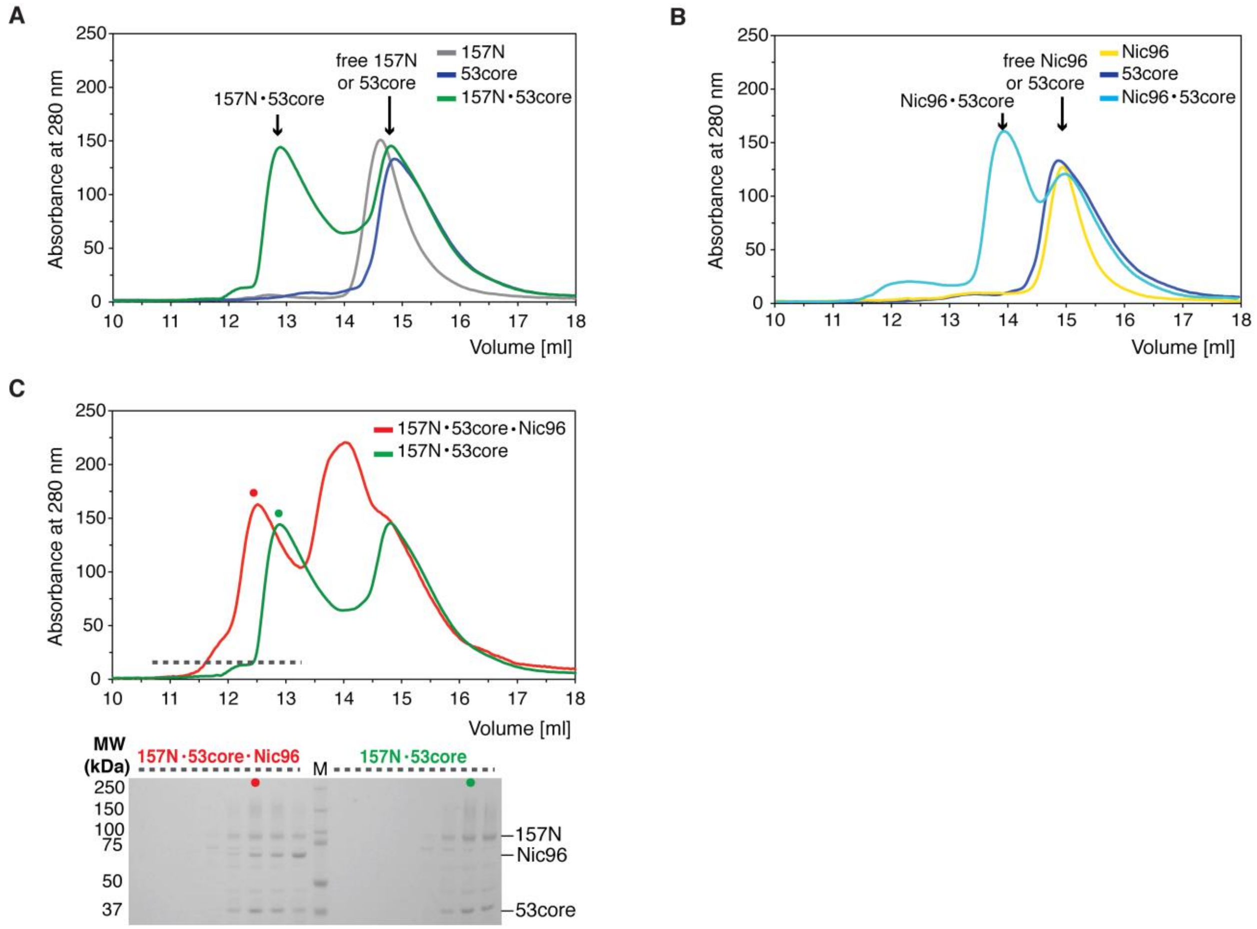

Fig. S6. Reconstitution of Nup53 into complexes with Nic96 and Nup157. Reconstitution of (**A**) 53core·157N, (**B**) 53core·Nic96, and (**C**) 157N·53core·Nic96 complexes by size-exclusion chromatography (SEC). The elution profiles of individual nups and their binary and ternary complexes are indicated with different colors. Gray bars indicate peak fractions that were resolved by SDS-PAGE (panel C).

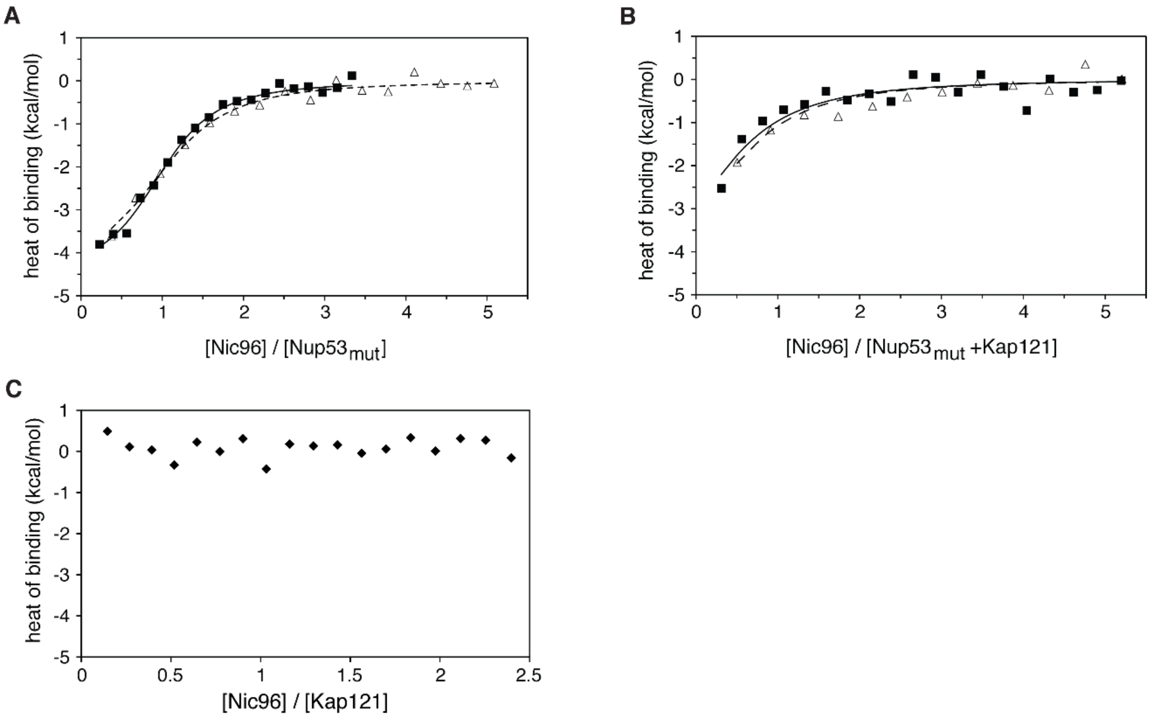

Fig. S7. Kap121 allosterically destabilizes Nic96 interactions with dimerization-deficient Nup53. (**A-C**) Representative binding isotherms obtained from the titration of (A) Nic96 at 325 μM (triangles) and 200 μM (squares) to dimerization-deficient Nup53$_{mut}$ at 10.6 μM and 10 μM, respectively, (B) Nic96 at 200 μM (triangles) and 258 μM (squares) to Nup53$_{mut}$ complex with Kap121 (each at 8 μM), and (C) Nic96 (190 μM) to Kap121 (15.5 μM), as monitored by ITC. Note that Nup53$_{mut}$ and 53core bind to Nic96 with comparable affinities (binding constants ~10^6 M^{-1}). Black squares and white triangles represent two independent titrations in (B) and (C).

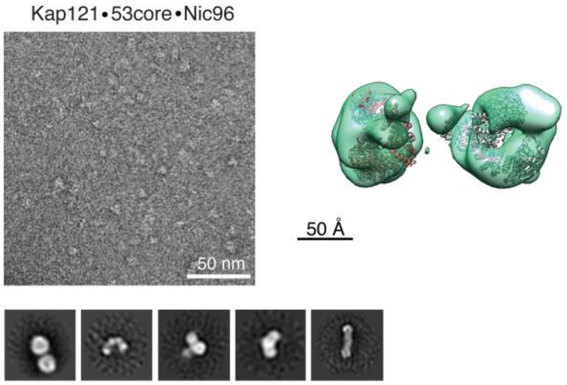

Fig. S8. NS-EM analysis of the Kap121·53core·Nic96 complex. A representative electron micrograph (top left panel) is shown with select 2D class averages (bottom panel). The 3D reconstruction of the dimeric conformation of the complex (top view) is displayed with a fitted crystal structure of Kap121 (PDB code: 3W3T; top right panel). Of note is that the electron density connecting the two Kap121 molecules is largely missing from the structure as compared to the same region in the Kap121·53core complex (for comparison see **Fig. 3F** and fig. S5A). Moreover, the precise location of the bound Nic96 in this 3D model could not be identified.

Table S1. Bacterial expression constructs.

**Protein***	**Residues**	**Expression vector**	**Expression conditions**
*Sc*Nup53	1-438	pGEX-4T1	18°C / 12-16 hours
*Sc*Nup53$_{mut}$ (F256E, W313E)	1-438	pGEX-4T1	18°C / 12-16 hours
*Sc*Nup53 (53NTE)	1-70	pGEX-4T1	18°C / 12-16 hours
*Sc*Nup53 (53N)	71-246	pGEX-4T1	18°C / 12-16 hours
*Sc*Nup53 (RRM)	247-355	pGEX-4T1	18°C / 12-16 hours
*Sc*Nup53 (53C)	357-438	pGEX-4T1	18°C / 12-16 hours
*Sc*Nup53 (53core)	151-438	pGEX-4T1	18°C / 12-16 hours
*Sc*Nup53 (53NTD)	151-246	pGEX-4T1	18°C / 12-16 hours
*Sc*Nup53 (53RRM-C)	247-438	pGEX-4T1	18°C / 12-16 hours
*Sc*Nup53 (53RRM-C*)	357-400	pGEX-4T1	18°C / 12-16 hours
*Sc*Nup53 (53CTD)	401-438	pGEX-4T1	18°C / 12-16 hours
*Sc*Nup53 (53CTDΔ)	417-438	pNIC28-Bsa4	18°C / 12-16 hours
*Sc*Nic96	189-839	pGEX-4T1	18°C / 12-16 hours
*Sc*Nup157 (157N)	1-893	pGEX-4T1	18°C / 12-16 hours
*Sc*Nup157 (157C)	900-1391	pGEX-4T1	18°C / 12-16 hours
*Sc*Kap121	1-1089	pNIC28-Bsa4	18°C / 12-16 hours
*Hs*Nup35 (*Hs*RRM)	170-250	pGEX-4T1	18°C / 12-16 hours
*Rn*Nup58 FG	416-530	pET28a	30°C / 3 hours
*Hs*Kapβ1	1-442	pGEX-6P1	30°C / 3 hours

*Uniprot accession codes for the proteins used in this study: *Sc*Nup53 (Q03790), *Sc*Nic96 (P34077), *Sc*Nic96 (P40064), Kap121 (also known as Pse1; P32337), *Hs*Nup35 (Q8NFH5-1), *Rn*Nup58 (P70581-1), *Hs*Kapβ1 (Q14974-1).

Table S2. Data collection and refinement statistics for the *Sc*Nup53 RRM domain (molecular replacement).

	**53-RRM** (5UAZ)
**Data collection**	
Wavelength (Å)	1.0000
Space group	$C222_1$
Cell dimensions	
a, *b*, *c* (Å)	41.50, 100.49, 85.23
α, β, γ (°)	90.0, 90.0, 90.0
Resolution (Å)	43.28 - 1.75 (1.82 - 1.75)[a]
R_{pim}	0.034 (0.334)
R_{merge}	0.119 (1.049)
$I/\sigma(I)$	21.7 (2.6)
Completeness (%)	99.9 (98.7)
Redundancy	13.3 (9.9)
Wilson B factor ($Å^2$)	25.4
**Refinement**	
Resolution (Å)	43.28 - 1.75 (1.82 - 1.75)
No. reflections	18,275 (1779)
R_{work} / R_{free}	0.156 / 0.198
No. atoms	
Protein	1509
Ligand/ion	8
Water	135
B factors ($Å^2$)	
Protein	35.4
Ligand/ion	68.3
Water	38.5
R.m.s. deviations	
Bond lengths (Å)	0.013
Bond angles (°)	1.25
Ramachandran statistics[b] (%)	
Favored/Allowed/Disallowed	99.4/0.6/0

Data that led to this structure were collected on a single crystal.
[a] Values in parentheses are for highest-resolution shell.
[b] As determined by MolProbity.
